# How does the structure of data impact cell–cell similarity? Evaluating how structural properties influence the performance of proximity metrics in single cell RNA-seq data

**DOI:** 10.1093/bib/bbac387

**Published:** 2022-09-23

**Authors:** Ebony Rose Watson, Ariane Mora, Atefeh Taherian Fard, Jessica Cara Mar

**Affiliations:** Australian Institute for Bioengineering and Nanotechnology, The University of Queensland, Brisbane, QLD, Australia; School of Chemistry and Molecular Biosciences, The University of Queensland, Brisbane, QLD, Australia; Australian Institute for Bioengineering and Nanotechnology, The University of Queensland, Brisbane, QLD, Australia; Australian Institute for Bioengineering and Nanotechnology, The University of Queensland, Brisbane, QLD, Australia

**Keywords:** single-cell RNA-seq, data structure, evaluation framework, similarity, distance, single cell clustering

## Abstract

Accurately identifying cell-populations is paramount to the quality of downstream analyses and overall interpretations of single-cell RNA-seq (scRNA-seq) datasets but remains a challenge. The quality of single-cell clustering depends on the proximity metric used to generate cell-to-cell distances. Accordingly, proximity metrics have been benchmarked for scRNA-seq clustering, typically with results averaged across datasets to identify a highest performing metric. However, the ‘best-performing’ metric varies between studies, with the performance differing significantly between datasets. This suggests that the unique structural properties of an scRNA-seq dataset, specific to the biological system under study, have a substantial impact on proximity metric performance. Previous benchmarking studies have omitted to factor the structural properties into their evaluations. To address this gap, we developed a framework for the in-depth evaluation of the performance of 17 proximity metrics with respect to core structural properties of scRNA-seq data, including sparsity, dimensionality, cell-population distribution and rarity. We find that clustering performance can be improved substantially by the selection of an appropriate proximity metric and neighbourhood size for the structural properties of a dataset, in addition to performing suitable pre-processing and dimensionality reduction. Furthermore, popular metrics such as Euclidean and Manhattan distance performed poorly in comparison to several lessor applied metrics, suggesting that the default metric for many scRNA-seq methods should be re-evaluated. Our findings highlight the critical nature of tailoring scRNA-seq analyses pipelines to the dataset under study and provide practical guidance for researchers looking to optimize cell-similarity search for the structural properties of their own data.

## Introduction

Single-cell RNA-sequencing (scRNA-seq) methods provide a means to investigate the heterogeneity of complex cell populations. High-resolution transcriptional profiles in scRNA-seq data can be used to discover signature genes and their expression that denotes specific cellular processes [1], states [2] and types [3]. Proximity metrics, such as Euclidean distance, are used to measure the cell–cell similarity of these transcriptional profiles, from which clustering algorithms attempt to identify sub-populations of cells within the dataset [4–6].

Cluster analysis of scRNA-seq data is challenging because of the way scRNA-seq data is structured. A primary example is the high rate of dropouts resulting in sparse and noisy datasets [7]. When paired with the capacity to measure thousands of features per cell, this sparsity results in increasingly high-dimensional (HD) data spaces with unique properties and limitations [8]. Furthermore, common clustering algorithms for scRNA-seqperform best when there are discrete groups of cells present in the data [4]. While these discretely structured datasets do exist (e.g. terminally differentiated cell-types) [9–11], datasets of continuous structure are also common. Continuously structured datasets are composed of contiguous groupings of cells which experience multifaceted gradients of gene expression, encompassing dynamic processes such as embryonic development [12, 13] and cell differentiation [14, 15]. Heiser and Lau [16] identified that a dataset’s structure is the primary determinant of dimensionality reduction (DR) performance, finding poorer preservation of structure in discrete datasets than in continuous ones. The assumption of discrete cell types in scRNA-seq clustering also poses challenges for identifying rare cell populations because rare cells may differ from more abundant, stable cell populations by only a small number of genes [17–19]. Despite their low abundance, rare cell populations are critically important because they often are highly specialized cell states or sub-types and therefore provide valuable insights into core processes such as differentiation, migration, metabolism and cancer [20–23]. It is also thought that the origin of a disease may be sourced to a subpopulation of cells or perhaps even a single cell. While this claim remains under debate, it emphasizes the importance of being able to confidently capture rare cell populations for clinical applications of scRNA-seq [24].

Similarities between cells based on gene expression are assessed using a proximity metric and this step forms the basis for all clustering algorithms. However, while the performance of clustering methods has been evaluated extensively with respect to structural properties of data [4, 5, 25–35], evaluation of which proximity metric to choose have remained limited, often producing varied recommendations and lacking key design considerations. For example, Skinnider *et al.* [36] recommend proportionality-based metrics, whilst Kim *et al.* [37] recommend correlation-based metrics, specifically Pearson. However, Sanchez-Taltavull *et al.* [38] recommend Bayesian correlation over Pearson. Despite the different findings of previous works with respect to specific proximity metrics, they are largely in agreement that metric performance is highly dataset-dependent [4, 37, 39–41]. This conclusion remains unworkable however, as the specific structural properties of the scRNA-seq datasets included in these evaluations are rarely addressed in detail or evaluated in a systematic manner.

Consequently, our study aims to address the important question of how the properties of scRNA-seq datasets influence the performance of proximity metrics (including true distance, correlation, proportionality, binary and dissimilarity measures) in scRNA-seq cell clustering. To the best of our knowledge, such an investigation has yet to be performed and may be a reason why previous attempts that have been more limited and unable to yield actionable conclusions. Our study evaluates the impact of 17 different proximity metrics on clustering performance for datasets that are Continuous and Discrete. Levels of cell-rarity, sparsity and dimensionality are varied to reflect the variability of scRNA-seq data. Our findings demonstrate that there are clear differences in the performance of these metrics depending on the structure of the data. Therefore, accounting for structural properties of the dataset when planning and executing an analysis pipeline leads to substantial improvements in performance. We believe similar performance gains may be possible in other parts of the analysis pipeline that depend on a proximity metric, such as DR and trajectory inference. Consequently, we provide readers with practical guidelines for selecting a preferred proximity metric and neighbourhood size with respect to the structural properties of their own datasets. Furthermore, our evaluation framework is available as a python package, *scProximitE*, to allow users to evaluate the performance of proximity metrics for their own datasets and structural properties of interest.

## Methods

### scRNA-seq data collection

A representative dataset was constructed for the Discrete structure from the CellSIUS benchmarking dataset [32, 42] and included cells from eight human cell lines. The Continuous structure category was represented by a subset of five erythrocyte differentiation cell types from the Fetal Liver Haematopoiesis dataset from Popescu *et al.* [43, 44]. For each dataset provided cell-type annotations were used as the ground truth to evaluate clustering performance (see Supplementary–Primary Analysis).

Within the Continuous and Discrete datasets, a subclass was defined to reflect the balance of cell-type proportions. A dataset is Abundant if the majority of cell populations are present at a relatively high level, specifically, a proportion of ≥5% of the total cell number. The first subset, Discrete-Abundant, contains seven cell lines at proportions of low (5.4%) to high (32%) abundance, and one moderately rare population (2%), whilst in the Continuous-Abundant dataset, all five cell populations were present at high proportions (20%). In contrast, a dataset is Rare if the majority of cell populations are at proportions of <5%. The Discrete-Rare dataset comprises of six rare cell populations (0.08–3.14%), and two highly abundant cell populations at 40.15 and 50.21%. The Continuous-Rare dataset consists of three rare cell types present at proportions between 0.075 and 2.5%, and two highly abundant populations (42%, 55%) (see Supplementary–Primary Analysis, Supplementary Table S1, see Supplementary Data available online at https://academic.oup.com/bib).

### scRNA-seq data simulations

Simulated datasets are used to evaluate how structural properties influence proximity metric performance, including sparsity and cell-population imbalance. The simulated datasets, produced with PROSST (v1.2.0) [45], follow a topology of four differentiation trajectories, diverging from a single origin state (Detailed in Supplementary–Primary Analysis). This dataset in its original form represents the Continuous-Abundant Simulated dataset, whilst a subset containing only the origin state and the endmost population from each differentiation path represents the Discrete-Abundant Simulated dataset (Supplementary Figures S1 and S2, see Supplementary Data available online at https://academic.oup.com/bib).

To further explore the influence of imbalanced cell-type proportions on metric performance, two structural subclasses, Rare and Ultra-Rare, were created using Continuous-Abundant and Discrete-Abundant simulated datasets. For the Rare dataset, multiple cell types are present at proportions *p* where 1% < *p* < 5%, while the Ultra-Rare datasets contain multiple cell types where *p* < 1% (see Supplementary Table S2, see Supplementary Data available online at https://academic.oup.com/bib). The final structural property of interest in the study is dataset sparsity. Starting at 46–50% sparsity, two additional levels, moderate (68–71%) and high (89–90%) sparsity, were produced for each of the six datasets by adding zeros using a Gaussian distribution (Supplementary Table S3,see Supplementary Data available online at https://academic.oup.com/bib).

### scRNA-seq data quality control and normalization

Raw count matrices were filtered to remove (i) cells with non-zero gene expression for <200 genes, (ii) cells with >10% of their total counts from mitochondrial genes and (iii) genes expressed in <10% of cells. The resulting cell and gene numbers for each dataset post-processing are in Supplementary Table S3 (simulations, see Supplementary Data available online at https://academic.oup.com/bib) and Supplementary Table S4 (CellSIUS and FSH, see Supplementary Data available online at https://academic.oup.com/bib). Gene expression measurements for each cell were normalized by total expression and multiplied by a scale factor of 10 000, log_e_-transformed, adding a pseudo count of one. All data processing steps, including filtering, normalization and identification of highly variable genes, were performed using Scanpy (v1.8.2) [46] (see Supplementary–Primary Analysis).

### Proximity metrics

A total of 17 proximity metrics with a diverse range of properties were evaluated (see Supplementary–Primary Analysis, Supplementary Table S5, see Supplementary Data available online at https://academic.oup.com/bib). True distance metrics are dissimilarities that satisfy four key properties of symmetry, reflexivity, non-negativity and the triangle inequality. This study included Euclidean, Manhattan, Canberra, Chebyshev and Hamming distances. Although the remaining 12 proximity measures do not strictly satisfy all properties of a distance metric, we refer to all as ‘proximity metrics’ herein for simplicity.

Hamming, Yule, Kulsinski and Jaccards Index are computed on binary vectors. To binarise the count matrices, 1 maps to genes with ≥1 count, and 0 maps to genes with zero expression. Several of the evaluated dissimilarities are derived from correlations: Pearson, Spearman, Kendall and Weighted-Rank. As scRNA-seq data is relative rather than absolute, two proportionality-based metrics were included: Bray-Curtis, a measure of compositional dissimilarity between two different samples, and Phi, which was found to perform well in scRNA-seq clustering [36]. Cosine measures the cosine of the angle between two vectors in multi-dimensional space.

In addition to commonly applied metrics, several recent scRNA-seq metrics were also included. Given the sparse nature of scRNA-seq data, we evaluated the Zero-Inflated Kendall correlation (ZI-Kendall), an adaptation of Kendall’s tau for zero-inflated continuous data. Additionally, we evaluated Optimal Transport (OT) distance with entropic regularization [47].

### Performance evaluation framework

ScRNA-seq datasets representing structural classes of interest were pre-processed and then input to calculate a distance matrix for each proximity metric. For each distance matrix, *k*-nearest-neighbour (KNN) graphs were then computed where each cell is connected to its *k* closest cells, as determined by the input distance matrix (see Supplementary–Primary Analysis). To account for varying degrees of local structure, KNN-graphs were constructed for each proximity metric at multiple neighbourhood sizes: 3, 10, 30 and 50. The resulting graphs are provided as input to the Scanpy implementation of the Leiden algorithm [48].

The Leiden algorithm identifies clusters as groups of cells that are more densely connected to each other than to the cells outside of the group based on the KNN-graph [48]. Leiden is an unsupervised method with a resolution parameter that can be tuned to influence the number of communities detected. To accomplish accurate benchmarking, the resolution parameter was adjusted automatically until the number of clusters in the ground-truth annotations was returned, or until 1000 iterations had been attempted. To account for initialisation bias, 10 random seed values were generated, and clustering was repeated with each seed for each KNN-graph (see Supplementary–Primary Analysis).

The performance of the individual clustering outputs for each KNN-graph was compared with ground-truth annotations and quantified using the Pair Sets Index (PSI) [49] implemented with genieclust (v1.0.0) [50] (see Supplementary–Primary Analysis). We also considered Adjusted Rand Index (ARI) [51] and Adjusted Mutual Information (AMI) [52] (see Supplementary–Primary Analysis) but PSI was the method of choice because any incorrect clustering of rare and abundant cell-populations affects this score equally. PSI has also been shown to be less sensitive to other clustering parameters such as the number of clusters and degree of cluster overlap [49]. PSI is a cluster validation metric based on pair-set matching and adjusted for chance, with a range of 0–1, where 0 indicates random partitioning whilst 1.0 represents perfect labelling with respect to ground truth annotations. The mean PSI across the clustering outputs was used to evaluate the neighbourhood size, *k*. Lastly, a mean PSI value was computed across the four neighbourhood sizes to summarize a proximity metric’s performance on a dataset.

## Results

**Figure 1:**
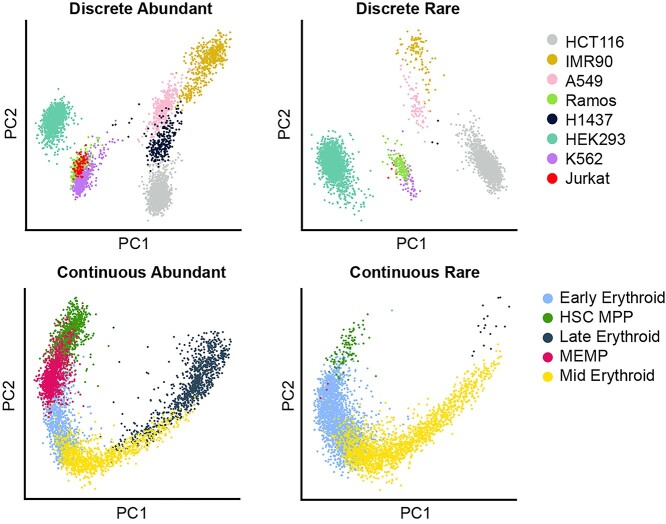
Principal Component Analysis (PCA) of the Discrete (top) and Continuous (bottom) datasets, from the CellSIUS and Fetal Liver Haematopoiesis datasets, respectively, subsampled to produce an Abundant (left) and Rare dataset (right).

**Figure 2:**
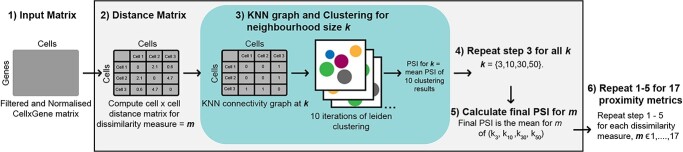
Evaluation framework for the assessment of clustering performance of proximity metrics (see Methods).

We developed our evaluation framework to assess how metrics performed based on properties relevant to scRNA-seq data (Figures 1 and 2). Specifically, the 17 proximity metrics were evaluated for four major types of scRNA-seq data structure: Discrete-Abundant, Discrete-Rare, Continuous-Abundant and Continuous-Rare. Additional to these structural classes, we evaluated the influence of (i) dimensionality, (ii) cell-population rarity, (iii) sparsity and (iv) neighbourhood density.

**Figure 3:**
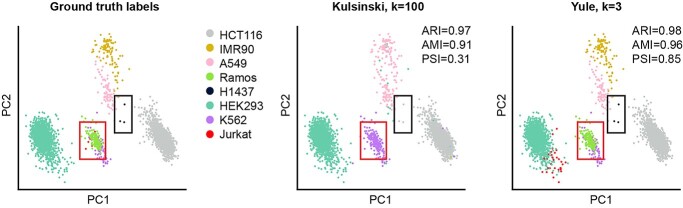
PCA in (**A**) depicts the ground-truth cell annotations for the Discrete-Rare dataset. PCA in (**B**) depicts the clustering results for the Discrete-Rare data with the Kulsinski metric at a neighbourhood size of *k* = 100. PCA in (**C**) depicts clustering results for the Discrete-Rare data with Yule at a neighbourhood size of *k* = 3. Boxes are included to emphasize the location of the rare cell-types H1437 (navy) and Jurkat (red) in each plot.

Comparisons to ground-truth cell annotations were assessed using PSI, ARI and AMI evaluation methods. We found the clustering score was dominated by the performance on Abundant populations, with little influence from Rare populations. For example, ARI and AMI scored a clustering output as near perfect on the Discrete-Rare dataset (0.97 and 0.91, respectively) despite six of the eight cell types being incorrectly clustered (Figure 3). Almost equivalent scores (ARI = 0.98, AMI = 0.96) were achieved by a clustering output where six of the eight cell types were accurately identified, showing the inability of these metrics to effectively distinguish clustering quality on datasets with substantial cluster-size imbalances. In comparison, PSI scored the second clustering result substantially higher (0.85) than the first (0.31).

### Clustering performance of proximity metrics is dependent on the intrinsic structure of scRNA-seq datasets

**Figure 4:**
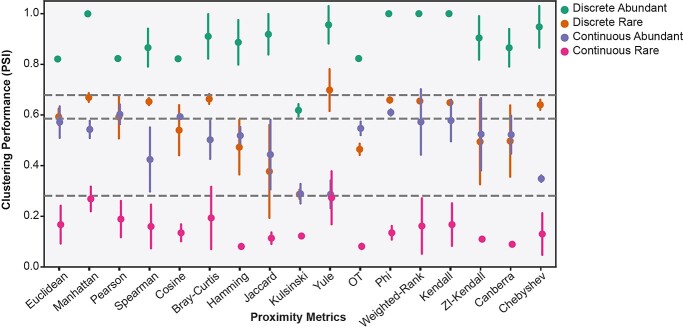
Clustering performance of proximity metrics for the scRNA-seq datasets representing the four classes of data structure: Discrete-Abundant, Discrete-Rare, Continuous-Abundant, Continuous-Rare. Points depict mean PSI of clustering from neighbourhood sizes of *k* = (3, 10, 30, 50), error bars depict one SD. Horizontal lines depict (top to bottom) 75th, 50th and 25th percentiles.

We find that the capacity of proximity metrics to identify similarities between cells correctly varies significantly depending on the intrinsic structure of scRNA-seq data (Figure 4). On average, proximity metrics achieved higher clustering performance for the Discrete data structures than the Continuous ones (on average by 0.4 PSI) (Figure 4). Within these structures, greater performance was observed for Abundant datasets than for Rare (average increase of 0.34 PSI) (Figure 4). The magnitude of differences in clustering performance was larger between dataset structures than between metrics evaluated within the same structure. For example, the standard deviation (SD) across all metrics within the Discrete-Abundant structure was only 0.097, while the SD for Euclidean distance across the four data structures was 0.27 (Figure 4). Similar trends are observed for simulated datasets and an additional four case-study datasets (Supplementary Figure S3, see Supplementary Data available online at https://academic.oup.com/bib).

### DR reliably improves clustering performance of proximity metrics in discretely structured datasets, but not continuously structured datasets

To evaluate how DR affects the performance of proximity metrics, we reduced the dimensionality by selecting the 2000 (HVG2000) and 500 most highly variable genes (HVG500) and compared their performance to the complete, HD datasets. Metrics were considered invariant between any two levels of dimensionality if there was <0.05 change in PSI.

**Figure 5:**
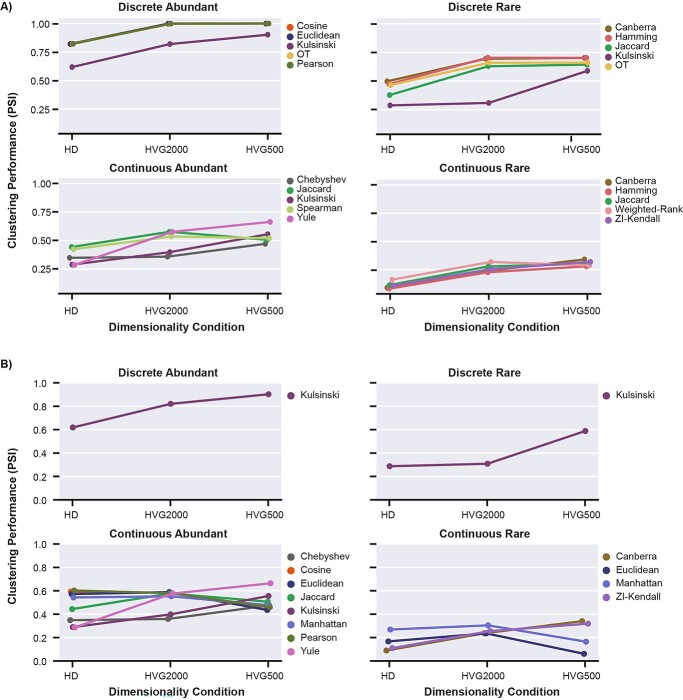
Clustering performance of (**A**) the top five metrics for each structural condition after ranking for greatest positive change between HD and either level of DR (HVG2000, HVG500), and (**B**) metrics with >0.05 change in PSI between HVG2000 and HVG500, for each structural condition. Each point represents PSI of clustering performance averaged across neighbourhood sizes (*k* = 3, 10, 30, 50).

As expected, an improvement in performance between the HD dataset and at least one of the HVG datasets was observed for a range of proximity metrics in all structural classes (Supplementary Figures S4 and S5, see Supplementary Data available online at https://academic.oup.com/bib). For Discrete-Abundant; Euclidean, Canberra, Hamming, Pearson, Spearman, Cosine and OT improved from <0.9 PSI to achieve near perfect clustering accuracy (>0.99 PSI) after DR (Figure 5A, Supplementary Figure S5, see Supplementary Data available online at https://academic.oup.com/bib). A similar trend was observed for Euclidean, Canberra and Hamming in Discrete-Rare, which ranked among the five highest performing metrics after DR to 500 HVG, despite relatively poor performance in HD (0.47–0.59 PSI) (Supplementary Figure S5, see Supplementary Data available online at https://academic.oup.com/bib). This indicates DR is of particular benefit to true distance metrics commonly applied in scRNA-seq analysis for discretely structured data.

Despite substantial improvements in clustering performance due to DR, metrics in Discrete-Rare data structures have lower PSI values (<0.71) than Discrete-Abundant structures. Similarly, when evaluating the Continuous data structures, the metrics with the largest improvement due to DR had overall lower PSI values than the Discrete structure: PSI <0.67 for Continuous-Abundant, and < 0.34 for Continuous-Rare (Figure 5A). Accordingly, the trends of poorer clustering performance with Continuous and/or Rare structure that are observed at HD largely remain after DR.

We next identify ‘robust’ metrics, characterized by a high level of performance and an invariant PSI across HD and HVG conditions. Such metrics may be an attractive option when performing DR is not feasible. We defined a high-performance metric as one with PSI at HD within 0.05 of the maximum PSI achieved for either level of DR within the corresponding dataset. Yule, Manhattan and Phi were identified as robust metrics for both Discrete datasets, along with Weighted-Rank for Discrete-Abundant (HD PSI >0.95) and Bray-Curtis for Discrete-Rare (HD PSI ≥0.66) (Supplementary Figure S6, see Supplementary Data available online at https://academic.oup.com/bib). Of the few proximity metrics identified as invariant for the Continuous-Abundant (3) and Continuous-Rare (4) datasets (Supplementary Figure S6, see Supplementary Data available online at https://academic.oup.com/bib), none were classified as high performing, indicating that DR has a greater influence on datasets with continuous structure and therefore is likely to be a necessary step prior to clustering.

Given the limited number of metrics showing invariance to DR on continuously structured data, we explored whether the extent of reduction applied (HVG2000 versus HVG500) influenced metric performance. Variable performance between the two HVG conditions was observed in approximately half the proximity metrics in Continuous-Abundant data (8/17) and a quarter in Continuous Rare (4/17) (Figure 5B). Euclidean and Manhattan were the only metrics that had a notable reduction in performance at HVG500 relative to HVG2000 for both continuous datasets. This contrasted with several other metrics which showed stronger performance with increasing DR.. In comparison, 16 of the 17 metrics in the Discrete datasets exhibited robust clustering performance between 2000HVG and 500HVG, with the outlier being Kulsinski (Figure 5B). This suggests that in discretely structured data, equivalent information may be captured with 500 genes as with 2000 for most metrics, but also that further reduction beyond 2000HVG does not provide additional benefits. Conversely, for continuously structured data there may be a narrower parameter range at which the benefits of DR are balanced with the loss of relevant structural information.

### All proximity metrics are sensitive to increasing rarity of cell-populations

To investigate if metric performance is only impacted beyond a certain rarity threshold, we generated Abundant (all populations >5%), Rare (multiple populations at >1 to <5%) and Ultra-Rare datasets (multiple populations at <1%) from simulated Continuous and Discrete data structures with moderate sparsity (68–71%) (Methods, Supplementary Table S2, see Supplementary Data available online at https://academic.oup.com/bib). Results for all sparsity levels are available in Supplementary Figure S7 (see Supplementary Data available online at https://academic.oup.com/bib).

**Figure 6:**
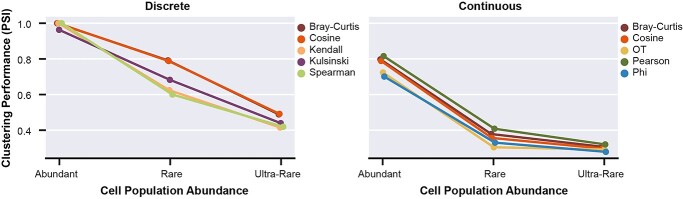
Clustering performance of the top five proximity metrics, as ranked by PSI on the Ultra-Rare subset (moderate sparsity), for Discrete (left) and Continuous (right) structured data. Points depict mean PSI of clustering from neighbourhoods of *k* = (3, 10, 30, 50). Error bars depict one SD.

Performance was substantially reduced between Abundant and Rare datasets for Discrete (0.29 mean change PSI=, SD = 0.09) and Continuous (0.24 mean change PSI, SD = 0.17), indicating that cell-populations at proportions ≥1% are sufficiently rare to challenge proximity metrics (Figure 6). Between the discretely structured Rare and Ultra-Rare datasets, performance was further reduced by a mean of 0.23 (SD = 0.07) across all metrics, with the maximum PSI of 0.49. There was no significant difference in PSI from Rare to Ultra-Rare datasets of Continuous structure (mean change in PSI = 0.04, SD = 0.03). This is unsurprising given that the metrics already displayed very poor performance for identifying Rare cell-types (≤0.41 PSI) (median PSI = 0.28, SD = 0.08). Notably, while Bray-Curtis and Cosine were among the top five performers for both Discrete and Continuous data structures based on PSI in Ultra-Rare datasets (Figure 6), all proximity metrics showed poorer performance with increasing rarity of cell-populations.

Our findings suggest that a threshold of ‘rarity’ (cell-population proportion) at which metric performance is suddenly impacted does not exist. Rather, we see a continuing decline in performance for cell populations of decreasing proportions relative to the total dataset. We show the metrics’ capacity to capture structural information is particularly challenged in datasets comprised of cell populations representing continuous processes and datasets containing rare cell populations.

### Most metrics have poorer performance as sparsity increases, but under-utilized metrics show greater robustness

Sparsity is one of the greatest challenges when working with scRNA-seq data and hence it is important to evaluate performance against this structural property. Therefore, we evaluated our Abundant and Rare simulated scRNA-seq datasets at three sparsity levels: low (46–50%), moderate (68–71%) and high (89–90%) (Methods). We defined a metric as robust to sparsity if the change between PSI levels for different sparsity conditions was ≤0.05, sensitive if the change between PSI levels was ≥75th percentile for all metrics in that structural class, and moderately sensitive if between these thresholds (Supplementary Figure S8, see Supplementary Data available online at https://academic.oup.com/bib).

**Figure 7:**
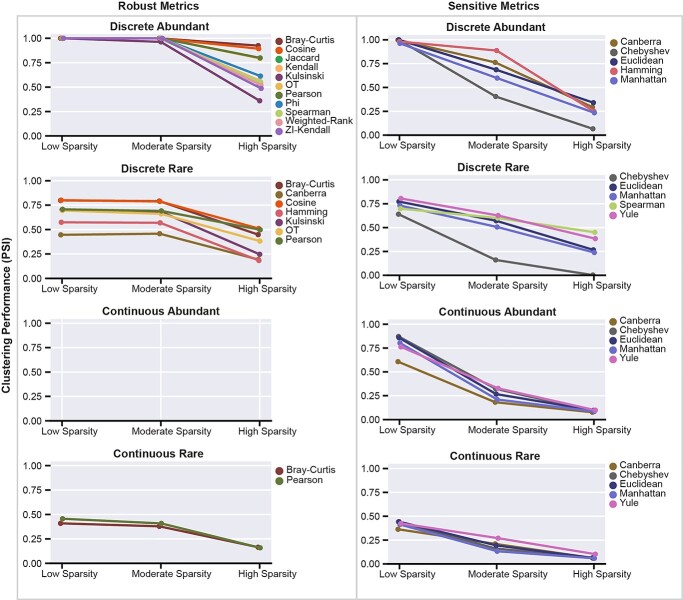
Left—Performance of proximity metrics identified as robust between low (50%) and moderate (70%) sparsity, given a threshold of ≤0.05 change in PSI. As no metrics met these criteria for the Continuous-Abundant dataset, the panel is blank. Right—Performance of proximity metrics identified as sensitive between low and moderate sparsity, given a threshold of ≥75th percentile change in PSI. Points depict mean PSI of clustering performance from simulated datasets across neighbourhoods of *k* = (3, 10, 30, 50).

Similar to DR, proximity metrics are influenced by sparsity to a greater degree on continuously structured data than on discretely structured data. Encouragingly, a substantial number of proximity metrics demonstrated robust performance when sparsity was increased from low to moderate for the Discrete-Abundant (11/17) and Rare (7/17) datasets (Figure 7). Conversely, no metrics were identified as robust for Continuous-Abundant, and only Bray-Curtis and Pearson correlation in Continuous-Rare. Notably, these were also identified as robust metrics for the discretely structured datasets. Furthermore, Bray-Curtis, Cosine and Pearson correlation were consistently ranked among the top five metrics with the least sensitivity to sparsity for all structural conditions (Supplementary Figure S9, see Supplementary Data available online at https://academic.oup.com/bib). However, it should be noted, the maximum PSI for the Continuous-Rare dataset with moderate sparsity was only 0.41, indicating that the clustering performance of even the best-ranked metrics was poor for this structure.

Interestingly, performance of the true distance metrics (Euclidean, Manhattan, Chebyshev and Canberra) was more sensitive to sparsity than other proximity metrics (Figure 7). Our results suggest that Bray-Curtis, Cosine and Pearson correlation may be the preferred metrics when analysing datasets with moderate sparsity levels, versus the more common Euclidean and Manhattan distance.

Despite maintaining clustering performance at moderate sparsity, all ‘robust’ metrics drop substantially in performance when applied to high sparsity data. Furthermore, at high sparsity, the performance for Abundant and Rare structures becomes equivalent in the Continuous dataset (maximum PSI 0.21) (Supplementary Figure S10, see Supplementary Data available online at https://academic.oup.com/bib). This indicates that insufficient information is present in highly sparse scRNA-seq data to enable the discrimination of contiguous cell-types, irrespective of cell-population abundance. The same trend is observed for the Discrete data, with the exception of Bray-Curtis, Cosine and Pearson correlation which provide good clustering performance for Abundant data (≥0.8 PSI). Consequently, reduction of sparsity is a key factor in optimizing performance of proximity metrics on scRNA-seq data, with particular necessity for continuously structured data.

### Dataset structure and sparsity are key factors in clustering parameter optimization

**Figure 8:**
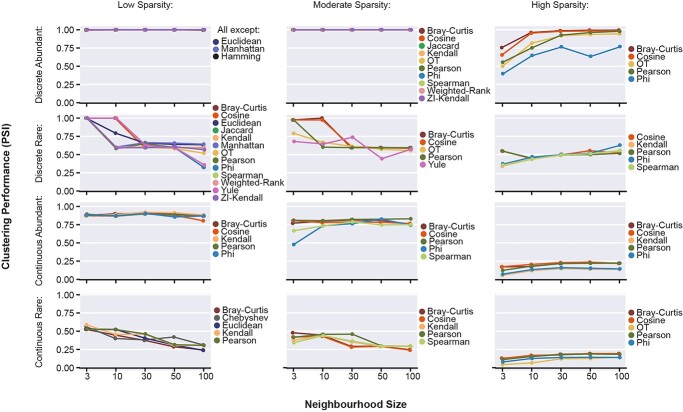
Clustering performance (PSI) (mean of *n* = 10 clustering iterations) across neighbourhood size values for KNN, for low sparsity (Left), moderate sparsity (middle) and high sparsity (right) simulations. Proximity metrics are included if their maximum PSI across all neighbourhood sizes is ≥75th percentile of the maximum performance in the relevant structural class.

For clustering approaches based on KNN-graphs such as the Leiden algorithm, the neighbourhood size of the graph, *k*, affects the number and size of clusters identified. We investigated the impact of neighbourhood size by varying *k* (*k =* 3, 10, 30, 50, 100) and evaluating metric performance for each simulated data structure and sparsity condition. To identify metrics with the strongest performance across all neighbourhood sizes, we focused on the maximum PSI value across all neighbourhood sizes ≥75th percentile (Figure 8).

At low sparsity, proximity metrics achieved greater performance at small neighbourhood sizes (3, 10) in Rare datasets of both Discrete and Continuous structure, whilst performance on Abundant datasets was invariant (Figure 8). These trends are weaker at moderate sparsity, as performance becomes more metric-specific. However, at high sparsity, metrics show increased performance at larger neighbourhood sizes (30, 50, 100) in the Discrete datasets, although in Discrete-Rare, Cosine and Correlation continue to exhibit greatest clustering performance at a neighbourhood size of 3. In the Continuous datasets, performance is consistently very poor regardless of neighbourhood size (<0.25 PSI). The inconsistent relationship between neighbourhood size and clustering performance at high-sparsity further underlines the challenges associated with capturing structural information from highly sparse scRNA-seq datasets and reinforces the recommendation to reduce dataset sparsity.

### Summary and practical recommendations

**Figure 9:**
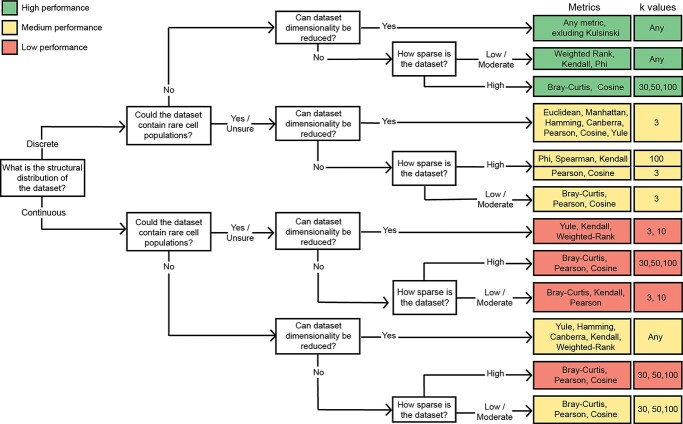
Flowchart for recommended metrics and neighbourhood sizes (*k*) given specific structural properties of an scRNA-seq dataset (Detailed in Supplementary Table S6, see Supplementary Data available online at https://academic.oup.com/bib). Proximity metrics recommended for ≥50% of structural conditions investigated include Pearson (8/12), Cosine (8/12), Bray-Curtis (7/12) and Kendall (6/12).

**Figure 10:**
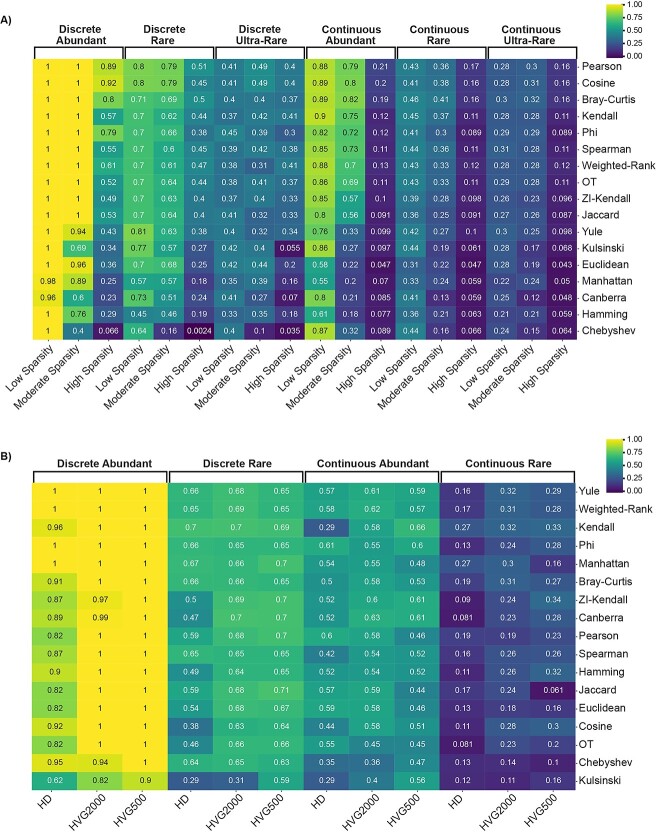
Proximity metric performance across real scRNA-seq datasets of varying structure and (**A**) sparsity and (**B**) dimensionality. Heatmap cells contain mean PSI obtained across all neighbourhood sizes. Rows are ordered by mean PSI across datasets with strongest performance at the top. Pearson, Cosine and Bray-Curtis showed the greatest robustness to dataset sparsity (**A**). Kendall correlation was among the top five metrics for both sparsity (**B**) and dimensionality (**A**), displaying a high degree of robustness relative to other metrics, whilst Euclidean distance exhibited equivalent or lower performance than a range of less common metrics across the dataset structures, showing sensitivity to both high-dimensionality (**B**) and sparsity (**A**). The adaptation of Kendall correlation for sparse data, ZI-Kendall, performed poorer than the original version under moderate and high sparsity conditions (**A**).

Our findings have been summarized in a flowchart to provide practical guidance on how to select an appropriate metric (Figure 9). Overall, the diverse nature of the metrics evaluated was exemplified in their differing responses to the structural properties investigated. For example, Cosine is the highest ranked metric for robustness to sparsity across all data structures (Figure 10A) but responded inconsistently to DR (Figure 10B). In contrast, Manhattan distance performance was robust to changes in dimensionality but is among the most sensitive metrics to even moderate sparsity.

When ranking metrics according to PSI at 30 neighbours only (the default value in Seurat), the top 5 ranked metrics remained the same for dimensionality, and top 4 metrics for sparsity, albeit re-ordered (Supplementary Figure S11, see Supplementary Data available online at https://academic.oup.com/bib). This suggests that our results may be relevant even without parameter tuning. To further evaluate the reliability of these recommendations, our framework was re-run on a new representative dataset for each structural condition: Discrete-Abundant [53], Discrete-Rare [54], Continuous-Abundant [55] and Continuous-Rare [56] (Supplementary–Validation Case Studies) (Supplementary Table S7, see Supplementary Data available online at https://academic.oup.com/bib). The top performing proximity metrics and neighbourhood sizes for these new datasets consistently aligned with those recommended for datasets of those structural properties in Figure 9 (Supplementary Figure S12, see Supplementary Data available online at https://academic.oup.com/bib). Furthermore, our case-study analysis demonstrates the robustness of our recommendations to additional variables introduced with these new datasets: different species (Human and Mice), multiple sequencing technologies (Drop-Seq, inDrops and 10x) and alternative pre-processing methods (scTransform [57]) (Supplementary–Validation Case Studies).

## Discussion

Given the direct influence of cell clustering on downstream analysis in scRNA-seq data, evaluating the accuracy of clustering algorithms is an important research area. Previous studies have recognized the effect of proximity metric choice when measuring cell–cell similarity on clustering performance [36, 37, 39]. However, variable performance is reported for proximity metrics between datasets, making the recommendation of a specific metric impossible [39]. In response, we developed a framework to evaluate 17 proximity metrics with respect to core structural properties of scRNA-seq data, including sparsity, dimensionality, structure and rarity. Our findings demonstrate that greater care should be taken to select and fine-tune methods to suit the structural properties of the individual dataset. Consequently, we have provided practical guidance for researchers to optimize their cell-similarity search by investigating and acting on the structural properties of their own data.

Of the actions available, we identified reducing dataset sparsity as the most impactful factor for improving clustering performance (Figure 7), whilst DR via selection of highly variable genes also produced improvements in clustering performance for many metrics (Figure 5). However, the variable results observed for continuously structured data indicate that the degree of DR must be tuned appropriately.

Selection of an appropriate neighbourhood size was essential for optimizing performance of metrics to accommodate cell-balance properties (Figure 8). Notably, the greatest performance for Rare datasets was obtained with neighbourhood sizes 3 and 10, versus the default values of 20 and 30 in Scanpy and Seurat, respectively. This illustrates the importance of tuning parameters for a given dataset based on knowledge of the underlying system, rather than relying on default settings [58]. Similarly, the optimal parameters for DR methods have been shown to be a function of dataset-specific properties [16, 59–61], and we expect that this extends to other scRNA-seq methods.

We consistently identified cell-population structure to be one of the most influential properties, with substantially lower clustering performance for metrics in continuously structured datasets than discretely structured (Figure 4, Supplementary Figure S3, see Supplementary Data available online at https://academic.oup.com/bib). This has previously been identified as a shortcoming of clustering methods, and alternatives such as pseudo-time analysis [62] or soft clustering [63] have been proposed [4]. However, given that these recommended alternatives similarly rely on the calculation of cell–cell similarity, selection of an appropriate proximity metric is equally relevant. Additionally, performance was inferior in datasets with imbalanced cell-population proportions due to rare cell-types, as compared to the Abundant datasets (Figures 4 and 6). While we identified preferred dataset processing steps, proximity metrics and parameter values to improve performance on Rare datasets (Figure 9), we were unable to match the clustering performance of the Abundant datasets for either Discrete or Continuous structures.

It is worth highlighting that only by using a performance score which is independent of cluster size, such as the PSI, could the true extent of this effect from rare cell populations be revealed (Figure 3) [49]. It is likely that unsatisfactory clustering accuracy due to rare cell populations is similarly present in other comparative evaluations but masked when using evaluation scores such as ARI and AMI. For ARI and AMI, cluster evaluations are size-dependent, and thus, the influence of misclassified rare cell populations on the overall score is greatly diminished [49, 64, 65]. Given common approaches for data processing, normalization, feature selection and clustering were used during our study, these findings raise concerns regarding the current state of rare cell-type identification in scRNA-seq. An extension to our work would be to include specialized clustering methods developed for rare cell-populations, such as GiniClust [33], scAIDE [34] or CellSIUS [32]. However, if researchers are unaware of the presence of rare cell types in their data, they may not seek out such specialized methods. As such, there is a crucial need for greater integration of rare cell-type methods into popular scRNA-seq packages and standard analysis.

Euclidean distance is among the most commonly applied metrics in scRNA-seq. Despite this, when evaluated for robustness to sparsity and high-dimensionality in our datasets Euclidean, and the other true distance metrics, showed greater sensitivity relative to some lesser known proximity metrics (Figure 7, Supplementary Figure S6, see Supplementary Data available online at https://academic.oup.com/bib). These results were not entirely unexpected, as true distances metrics can perform poorly as dimensionality and sparsity increase, leading to poorly defined nearest neighbours [66, 67]. In line with this, we saw true distance metrics perform considerably better with the appropriate level of DR, at times even achieving maximum performance (Figure 5).

Our findings support previous studies which have similarly identified Euclidean as a poorly performing proximity metric in scRNA-seq [36, 37, 39]. In Kim *et al.* [37] correlation-based metrics outperformed Euclidean distance for clustering, which was attributed to the sensitivity of the true distance metrics to scaling and normalization, whereas correlation-based metrics are invariant to these factors. Interestingly, Pearson and Kendall correlations, along with another scale-invariant metric, Cosine, were preferred metrics for the majority of structural conditions examined in our study. However, other scale-invariant metrics such as Spearman correlation did not show the same performance trends. Skinnider *et al.* [36] also found Euclidean performed poorly and suggested that as scRNA-seq only yields relative gene expression rather than an absolute, proportionality metrics such as Phi and Rho are more suitable [68]. Whilst Phi had moderate performance in our evaluation, it was outperformed by Pearson, Kendall and Cosine. However, another proportionality-based metric, Bray-Curtis, was a preferred metric for over half of the structural condition combinations evaluated.

Accordingly, in scenarios where cell-type annotations are unknown, users will have greater success identifying true cell groupings when using an alternative proximity metric that is suited to the structural properties of their dataset, as opposed to the default of Euclidean provided in most scRNA-seq analysis tools. Several clustering methods that use alternative metrics have already been shown to perform well for scRNA-seq data. For example, SC3 generates a consensus distance matrix derived from the Euclidean, Pearson and Spearman proximity metrics [69]. RaceID3 is a rare cell-type clustering method, which allows the user to select from a range of distance and correlation-based metrics [70]. Other methods have instead developed new metrics to measure cell–cell similarity, such as CIDER which recently proposed Inter-group Differential ExpRession (IDER) as a metric for their new clustering pipeline [31].

Our framework could be extended to include clustering methods beyond graph-based clustering. However, similar results were obtained for proximity metrics clustering performance by Skinnider *et al.* [36] when they compared hierarchical and graph-based clustering, suggesting that our results may hold for other methods. As with clustering, many scRNA-seq DR methods rely on the calculation of cell–cell similarity with a proximity metric. To minimize the influence of additional proximity calculations on the downstream clustering result, we used a feature-selection approach when exploring this aspect of data structure. However, given the popularity of alternative DR methods in scRNA-seq pipelines, such as PCA [71], t-SNE [72] and UMAP [73], an interesting future direction would be to investigate approaches based on feature transformation.

Furthermore, as these DR methods typically use Euclidean distance, the application of our framework to explore the influence of alternative proximity metrics on DR performance may prove insightful [74, 75]. Whilst consistent results were achieved with two different processing pipelines in this study (Supplementary–Validation Case Studies), we expect proximity metric performance to be impacted to some extent by dataset processing. Therefore, future extensions to the framework design to study the influence of pre-processing could be explored.

Taken together, our findings demonstrate how the inherent structural properties of scRNA-seq data have a substantial influence on the performance of proximity metrics and, resultantly, cell-type clustering and subsequent identification. Given the complexity of scRNA-seq datasets, it is unlikely for a single metric to perform best in all situations. Instead, we have provided practical guidelines for the selection of proximity metrics likely to perform well with respect to specific properties of the dataset. Furthermore, we provide our framework in the form of a python package to allow users to evaluate proximity metrics for their own datasets. The relevance of this study extends beyond cell clustering, to the numerous scRNA-seq analysis methods which make use of cell-to-cell distances. The findings from our study are expected to contribute to improvements in novel metric development for HD, sparse data such as scRNA-seq.

**Key Points**

- We developed a framework to systematically evaluate the influence of scRNA-seq data structural properties on the clustering performance of proximity metrics.
- Clustering performance can be improved substantially by selection of an appropriate proximity metric and neighbourhood size for the structural properties of a given dataset.
- Clustering performance for many proximity metrics was improved by reducing dataset sparsity and/or dimensionality.
- Popular metrics such as Euclidean distance performed poorly relative to lessor applied metrics including Cosine, Bray-Curtis and Pearson and Kendall correlations.
- Clustering accuracy with respect to rare cell populations is ineffectively evaluated by ARI and AMI due to their sensitivity to cluster size, and we recommend using size-independent metrics such as the Pair Sets Index for situations where bias based on cluster size is not useful.

## Supplementary Material

Supplementary_Resubmission_Jul26_bbac387

## Data Availability

The CellSIUS dataset [42] is available in Zenodo: https://zenodo.org/record/3238275. The Fetal Liver Haematopoiesis dataset [44] is available from the Developmental Human Cell Atlas: https://developmentcellatlas.ncl.ac.uk/datasets/hca_liver/data_share/. Our results, along with raw and processed copies of all datasets used are available at https://doi.org/10.5281/zenodo.6443267. The evaluation framework package scProximitE and code to reproduce all figures is available at https://github.com/Ebony-Watson/scProximitE.

## Author Biographies

**Ebony Rose Watson** is a PhD student from the Australian Institute for Bioengineering and Nanotechnology at the University of Queensland. Her research interests include identification and characterisation of rare cell-types in single-cell omics and imaging data.

**Ariane Mora** is a PhD student at the University of Queensland, Australia, and studies how epigenetics influence cell fate. Ariane researches data integration methods with a specific interest in representation learning.

**Atefeh Taherian Fard** is a postdoctoral researcher at the Australian Institute for Bioengineering and Nanotechnology at the University of Queensland. Her research interests include bioinformatics, transcriptomics, single cell and systems biology.

**Jessica Cara Mar** is an associate professor and group leader at the Australian Institute for Bioengineering and Nanotechnology at the University of Queensland. The group is focused on modeling variability of gene expression to understand how this regulates cellular phenotypes.
