# Supplementary material for: How does the structure of data impact cell–cell similarity? Evaluating how structural properties influence the performance of proximity metrics in single cell RNA-seq data
Source: Brief Bioinform. 2022 Sep 23;23(6):bbac387. doi: 10.1093/bib/bbac387 (PMC9677483; doi:10.1093/bib/bbac387)
Supplement: Supplementary_Resubmission_Jul26_bbac387 [file supplementary_resubmission_jul26_bbac387.docx]

# **How does the structure of data impact cell-cell similarity? Evaluating how structural properties influence the performance of proximity metrics in single cell RNA-seq data**

# **Supplementary Files**

**Primary Analysis**

**Methods**

*scRNA-seq Data Collection*

The CellSIUS benchmarking dataset [1,2] and the Fetal Liver Haematopoiesis dataset [3,4], representing the Discrete and Continuous structures respectively, were both produced using 10x Genomics Chromium Single Cell (3′ library kit V2). The provided cell-type annotations were used as the ground truth when evaluating clustering performance. For CellSIUS, single-cell sequencing was performed on batches of 2-3 cell-lines at a time, whilst bulk-sequencing was also performed for each cell line individually [1]. Cell-type annotations were generated by correlating single-cell profiles to the bulk profiles. For the FLH dataset, cell annotation was performed manually and validated through imaging mass cytometry, flow cytometry and cellular morphology [3].

The CellSIUS dataset was subset to create the Abundant and Rare subclasses as described by Wegmann *et al*. [1] (code available at Zenodo [2]) in R (v4.1.1) [5]. The resulting Seurat [6] objects were converted to Anndata [7] objects using the *sceasy* package (v0.06) [8] for subsequent analysis in Python (v3.8.11) [9]. To produce continuously-structured cell-populations, the Fetal Liver Haematopoiesis dataset [4] was subset to retain only cells labelled as 'Early Erythroid', 'Mid Erythroid', 'Late Erythroid', 'MEMP', and 'HSC_MPP'. These cell-populations were then reduced to the desired proportions by random subsampling with *Scanpy.preprocressing.subsampling* (v1.8.2) [10] (code available at *scProximitE* GitHub [11]). Details pertaining to cluster size and proportions for each dataset are available in Supplementary Table 1, and raw copies of all datasets are available at our Zenodo [12].

*scRNA-seq Data Simulations*

The simulated dataset follows a topology of four differentiation trajectories of equal length (3 branches), which diverge from a single origin state (1 branch). Each branch length is 50 pseudo-time units and represents a cell population in a continuous differentiation process. The *PROSST* package (v1.2.0) [13] was used to simulate 10 cells at each pseudo-time unit (6500 cells total) and 5000 genes from a negative binomial distribution. For each gene *g*, the variance parameters were sampled from α*_g_* ∼ e^x^, x ∈ N (log_e_ (0.2), 1.5) and β*_g_* ∼ e^x^, x ∈ N (log_e_ (1), 1.5), respectively. This simulated dataset in its original form was used to represent the Continuous-Abundant Simulated dataset, whilst a subset containing only the origin state and the endmost population from each differentiation path was used to represent the Discrete-Abundant Simulated dataset (Figure S1, S2). The Rare and Ultra-Rare subclasses for each structure were produced by random subsampling of the corresponding Abundant dataset with Scanpy (v1.8.2) [10] to the desired proportions (Supplementary Table 2). Code for generation and processing of the simulated datasets, including additional sparsity levels, is available at the scProximitE GitHub [11] and raw copies of all simulated datasets are available at our Zenodo [12].

*scRNA-seq Data Quality Control and Normalisation*

For consistency, all datasets were converted to Compressed Sparse Row matrix format using *sparse.csr_matrix* from Scipy (1.7.1) [14] prior to filtering and normalisation. All processing and quality control was conducted using the *preprocessing* module of Scanpy (v1.8.2) [10]. Quality control and metrics were computed with *calculate_qc_metrics* and datasets subset to remove cells with >10% of their total counts arising from mitochondrial genes (*pct_counts_mt* variable). The *filter_cells* and *filter_genes* functions were used to remove cells with non-zero gene expression for <200 genes and to remove genes expressed in <10% of cells, respectively. After filtering, the *normalise_total* function with *target_sum = 1e4* was applied, and log transformation performed with *log1p*. For the dimensionality reduction investigation, the *highly_variable_genes* function was applied to the processed datasets with *n_top_genes=2000* and *n_top_genes=500* to produce the HVG2000 and HVG500 datasets respectively. The resulting cell and gene numbers for each dataset post-processing are in Supplementary Tables 3 (simulations) and 4 (CellSIUS and FSH). Code for processing of all datasets is available at the scProximitE GitHub [11] and processed copies of all datasets are available at our Zenodo [12].

*Proximity Metrics*

Details pertaining to the class, input and implementation of each metric are provided in Supplementary Table 5. The formula for each proximity metric is available via the documentation of the relevant package. Distance matrices for proximity metrics implemented in python were computed using *scProximitE.proximity_matrix*. For proximity metrics implemented in the dismay [15] package in R (v4.1.1) [5], Anndata [7] objects were converted to Seurat [6] objects using the sceasy package (v0.06) [8]. Computed distance matrices were then exported from R in .csv format and, using *scProximitE.merge_metrics,* merged into the corresponding datasets Anndata object in Python (v3.8.11) [9] for subsequent analysis. The *merge_metrics* function automates conversion of the similarity matrices returned by dismay into symmetric dissimilarity matrices with a zero-diagonal for analysis. Code is available at the scProximitE GitHub [11].

*Performance Evaluation Framework*

The performance evaluation framework was developed in in Python (3.8.11) [58] (Figure 2). After computation of distance matrices, *k*-nearest-neighbour (KNN) graphs were computed using *neighbors.kneighbors_graph* from Scikit-learn (v1.0.1) [16] with *metric=‘precomputed’,* and *k = n,* for  *n* = (3, 10, 30, 50). Clustering was performed using *scProximitE.clustering* with *method=‘leiden’*, which employs the Scanpy (v1.8.2) [10] implementation of the Leiden Algorithm [17]. To accomplish accurate benchmarking, *scProximitE.clustering* adjusts the resolution parameter automatically until the number of clusters returned matches the number of cell-types in the provided annotations, or until 1000 iterations have been attempted. To account for initialisation bias in this study, 10 random seed values were generated using *numpy.random.randint* and Leiden clustering was repeated with each seed for each connectivity graph.

Clustering results were obtained for ≥ 9 of the 10 repeats for all proximity metrics from the CellSIUS and FLH datasets, apart from Kulsinski at *k* = 50 in the Discrete-Abundant Dataset, which obtained 7. For all simulation datasets, ≥ 7 of the 10 repeats were returned, with two exceptions. For Cosine in the high sparsity Discrete-Rare dataset at *k* = 100 only 4 results were obtained, and for Canberra in the high sparsity Continuous-Rare dataset at *k* = 3, where only 1 result was returned. Clustering performance was compared ground-truth cell annotations and quantified using the genieclust implementation (v1.0.0) [18] of the Pair Sets Index (PSI) [19]. The Scikit-learn (v1.0.1) [16] implementations of the Adjusted Rand Index (ARI) [20] and Adjusted Mutual Information (AMI) [21] were used to trial these as alternative evaluation scores (Figure 3). All evaluation metrics were called via the *scProximitE.cluster_stats* wrapper. The mean PSI across the clustering outputs for each *k* was computed using *pandas.Series.mean*, via the *scProximitE.load_pickle* function with *mode=‘mean’*.

Code for the complete evaluation framework and generation of all manuscript figures is available at the scProximitE GitHub [11], and results from the analysis are available at our Zenodo [12].

**Results**

In Discrete-Abundant datasets, application of DR ensured equally exceptional clustering performance (>99%) for all metrics and neighbourhood sizes, excluding Kulsinski (Figure 10B). Similar performance could be achieved even in highly sparse (Figure 10A) or high-dimensional datasets (Figure 10B), given appropriate selection of metric and neighbourhood size (Figure 9). For Discretely-Rare datasets, Pearson correlation and Cosine were top performing metrics at k=3 for all combinations of data properties (Figure 9, Figure 10). However, the greatest performance (0.71) could only be achieved in datasets which underwent DR (Figure 10B) or had low-moderate sparsity (Figure10A), highlighting the importance of data-processing prior to clustering. Application of DR also expanded the pool of high performing metrics to include many of the distance metrics and the binary metric, Yule (Figure 10B).

For Continuous structures, Yule, Kendall, and Weighted-Rank were consistently among the top performing metrics for Abundant and Rare datasets after application of DR, whereas the performance for some other metrics suffered upon DR to HVG500 (Figure 10B). In scenarios where datasets are unable to undergo DR, Pearson correlation, Bray-Curtis and Cosine were the highest performing metrics across all sparsity levels, although performance was significantly lower in the high sparsity datasets (Figure 10A).

**Validation Case-Studies Analysis**

To evaluate the reliability of our recommendations (Manuscript Fig. 9), a new representative dataset was sourced for each structural condition (Discrete-Abundant, Discrete-Rare, Continuous-Abundant, Continuous-Rare). To evaluate the robustness of the recommendations to common variations in scRNA-seq datasets, we included datasets from multiple species (Human and Mice), sequenced with different technologies (Drop-Seq, inDrops and 10x), and employed an alternative pre-processing approach to our original analysis.

**Methods**

*Dataset collection*

The Discrete-Abundant case study dataset was generated from a mouse retinal bipolar cell dataset produced by Shekhar *et al.* (2016) using droplet-based scRNA-seq (Drop-Seq) [23]. From the complete dataset, 6 cell types (Rod Bipolar cells, Cone Bipolar Cells type 2, 4, 6 and 7, Mueller Glia) were selected and subsampled to the proportions in Supplementary table 7.

The Discrete-Rare case study dataset was generated from a dataset of mice colon-derived stromal cells produced by Jasso *et al*. (2022) using the Chromium Single-Cell 3′ Gene Expression kit (v2, 10x Genomics) [24]. From the control dataset, 7 cell types (Fibroblast 1b, Glial, Interstitial Cells of Cajal 1, Lymphatic Endothelial Cells, Mesenchymal Stromal Cells, Pericytes, Smooth Muscle Cell 1) were selected and subsampled to the proportions in Supplementary table 7.

The Continuous-Abundant case study dataset was generated from a dataset of mouse bone marrow stromal cells produced by Wolock *et al*. (2019) using 3′ droplet-based scRNA-seq (inDrops) [25]. From the complete dataset, 5 cell types (Adipocyte progenitor, Mesenchymal Stromal Cells, Osteoblast progenitor, Pre-adipocyte, Pro-osteoblast) were selected and subsampled to the proportions in Supplementary table 7.

The Continuous-Rare case study dataset was generated from a dataset of human embryonic and fetal corneas produced by Collin *et al*. (2021) using Chromium Single Cell 3′ Library & Gel Bead Kit, version 3 (10x Genomics)[26]. From the week 13 and week 14 fetal datasets, 6 cell types (corneal stroma keratocytes type 1, 2 and 3, Fibroblasts type 1 and 2, and Neural Crest cells) were selected and subsampled to the proportions in Supplementary table 7.

*Data Processing and Analysis*

Filtering was conducted as described for the original datasets in the Primary Analysis Methods. After filtering, the Anndata object for each dataset was converted to a Seurat objects using the sceasy package (v0.06). Normalisation and correction of batch effects was performed using Seurats SCTransform function. The *return.only.var.genes* parameter was set to FALSE to ensure all genes were returned, and *vars.to.regress* set to include mitochondrial percentage, and the relevant biological sample and batch ID for each dataset [27]. Analysis with the scProximitE framework was then conducted as described in the manuscript and Primary Analysis Methods. Due to time constraints and results of the primary analysis, the Optimal Transport metric was excluded from this analysis.

**Results**

As observed in our primary analysis (Figure 4, Figure S3A), proximity metrics achieved higher clustering performance for the Discrete data structures than the Continuous, and within these structures greater performance was observed for Abundant datasets than for Rare (Figure S3B).

In line with the primary analysis, to identify metrics with the strongest performance across all neighbourhood sizes we focused on the maximum PSI value across all neighbourhood sizes ≥ 75th percentile (Figure S12). We then sought to verify if the top performing metrics and neighbourhood sizes for these datasets corresponded with the recommendations made in Figure 9, given their structural properties.

The Discrete-Abundant dataset had a sparsity of 78.93%, which sat between our ‘Moderate’ and High’ sparsity classes explored in the primary analysis, and interestingly metrics recommended for both high-sparsity (Bray-Curtis, Cosine) and low/moderate-sparsity DA datasets (Weighted-Rank, Kendall) were present in the 75% percentile (Figure S12A). Performance was also consistent across all neighbourhood sizes, as recommended for low/moderate-sparsity DA datasets. The Discrete-Rare dataset had a sparsity of 73.13%, within our low/moderate class, which suggested Bray-Curtis, Pearson or Cosine as top performers, at a neighbourhood size of 3. These recommendations were found to be the highest scoring metric and neighbourhood size combinations on this Discrete-Rare test-case as well (Figure S12B).

The Continuous-Abundant dataset had a sparsity of 78.14 %, again straddling the ‘Moderate’ and High’ sparsity classes from the primary analysis. For either class however, Bray-Curtis, Pearson and Cosine are recommended, at larger neighbourhood sizes (30,50,100), which were identified as the best-performing combinations for this Continuous-Abundant test-case as well (Figure S12C). The Continuous-Rare dataset had a sparsity of 71.51%, corresponding to our low/moderate class. Bray-Curtis and Pearson were both top-performers at a neighbourhood size of 3 (Figure S12D), in alignment with the recommendations made in Figure 9.

# **Supplementary Figures**


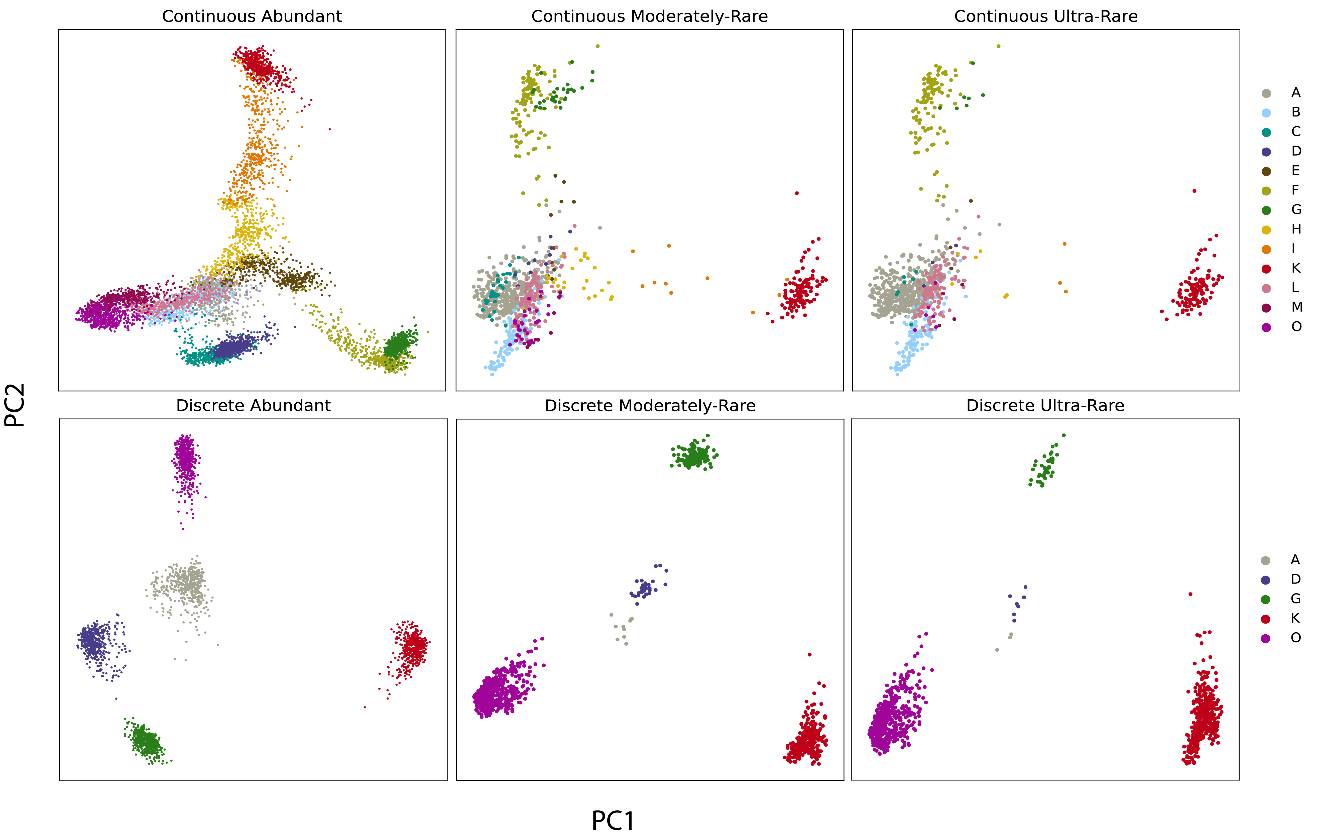


Discrete Abundant

Continuous Abundant

Discrete Rare

Continuous Rare

Discrete Ultra-Rare

Continuous Ultra-Rare

#

# **Figure S1:** Principal Components Analysis (PCA) on normalised and scaled data from the Continuous (top) and Discrete (bottom) simulated scRNA-seq data structures. Rows show the Abundant (left), Rare (centre), and Ultra-Rare (right) subsets of each structure. Cells are coloured by branch segment, representing the individual cell populations to be identified.

**Figure S2:** Principal Components Analysis (PCA) on normalised and scaled data from the Continuous (top) and Discrete (bottom) simulated scRNA-seq data structures. Rows show the Abundant (left), Rare (centre), and Ultra-Rare (right) subsets of each structure. Cells are coloured by pseudo-time, representing the distance in pseudo-time units of each cell from the origin (0).


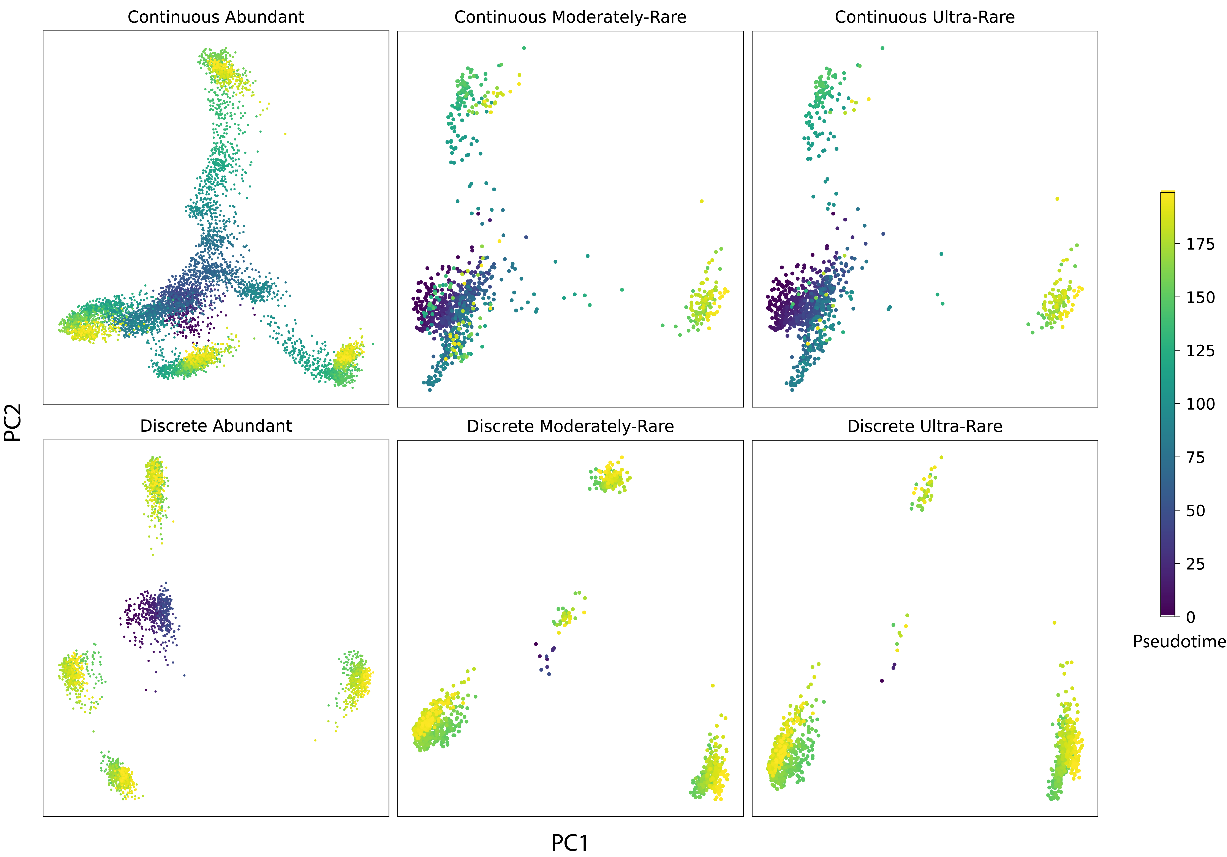


Continuous Abundant

Continuous Rare

Continuous Ultra-Rare

Discrete Abundant

Discrete Rare

Discrete Ultra-Rare


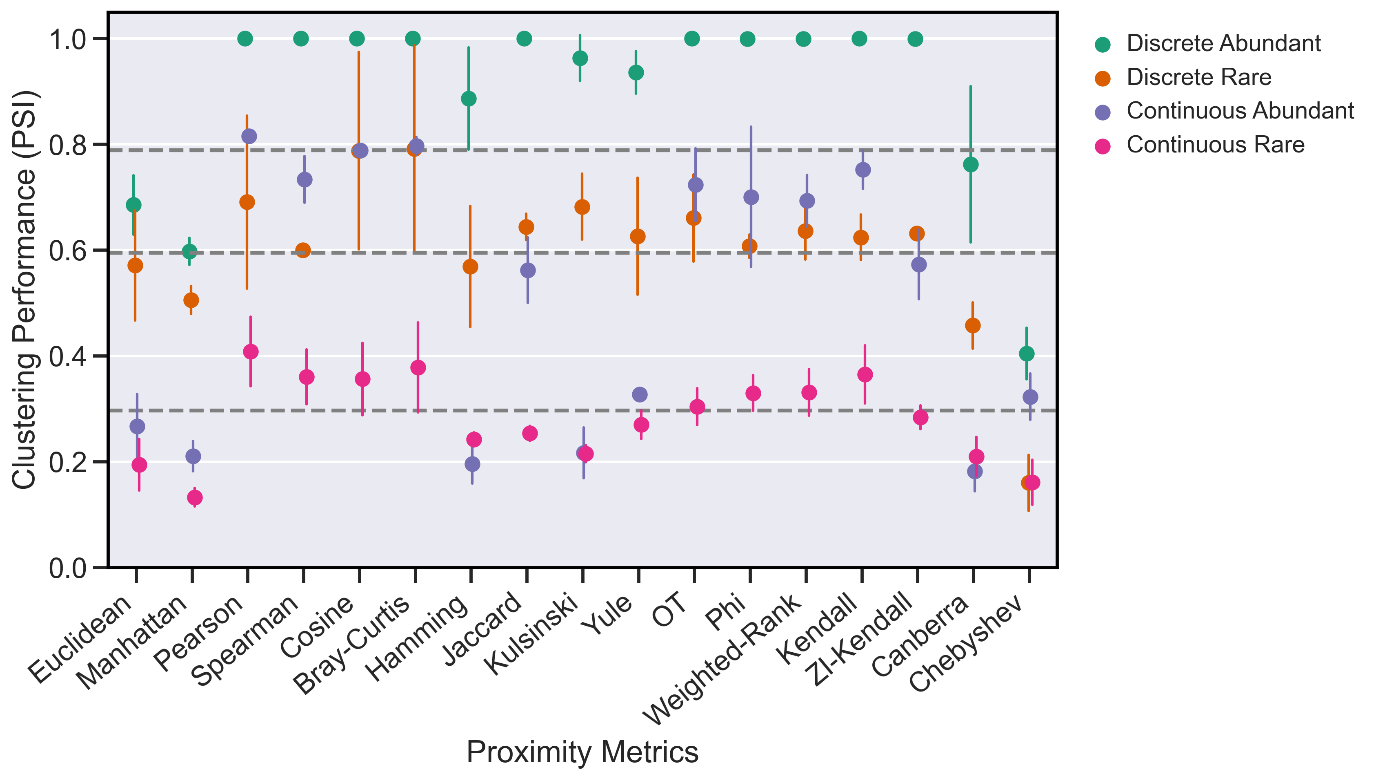

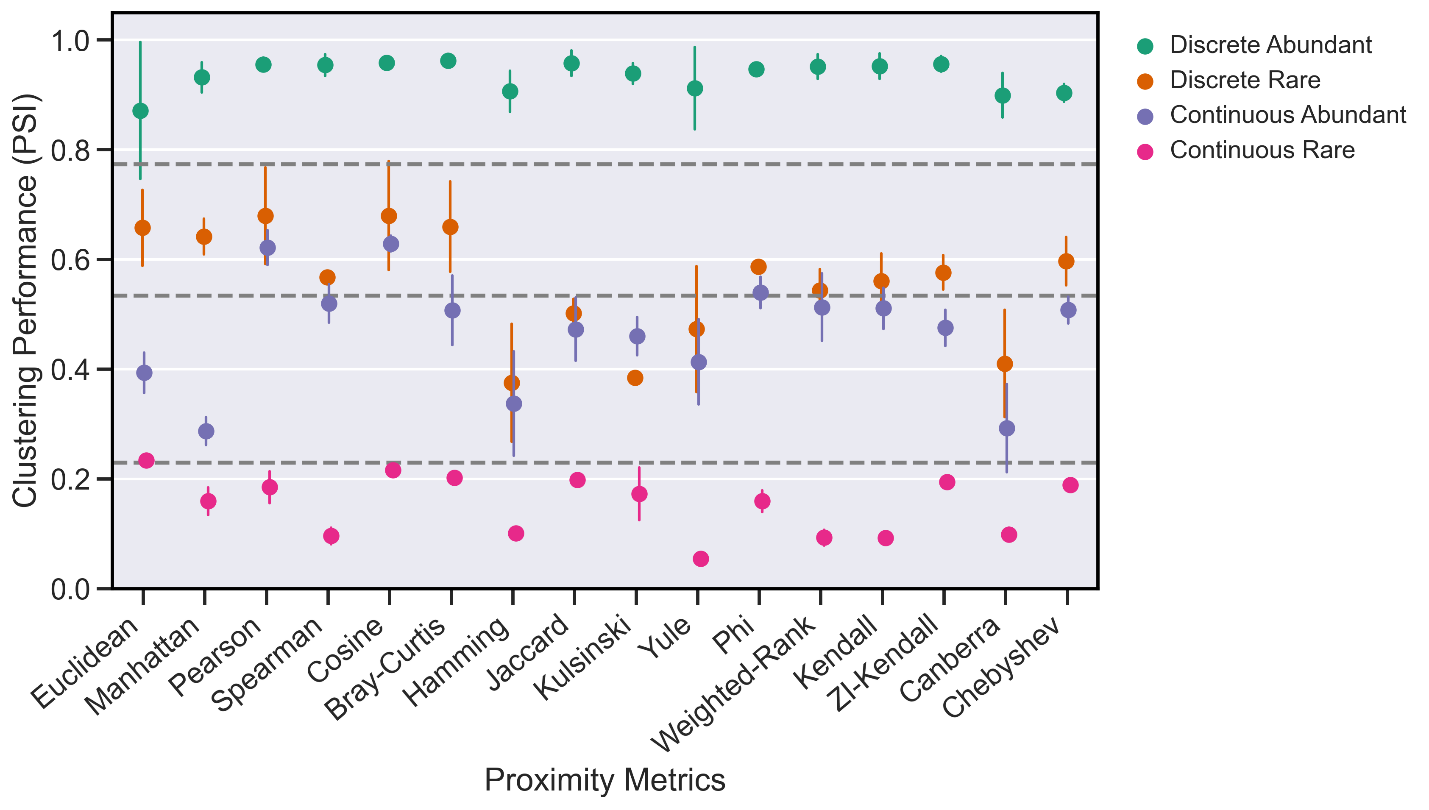


**A)**

**B)**

**Figure S3:** Clustering performance of proximity metrics for the **A)** simulated scRNA-seq datasets (moderate sparsity) and **B)** validation case-study datasets, representing the four classes of data structure: Discrete Abundant, Discrete Rare, Continuous Abundant, Continuous Rare. Points depict the mean Pair Sets Index (PSI) of clustering from neighbourhood sizes of *k* = (3,10,30,50), with error bars depicting one standard deviation. Horizontal lines depict (top to bottom) 75th, 50th and 25th percentiles.


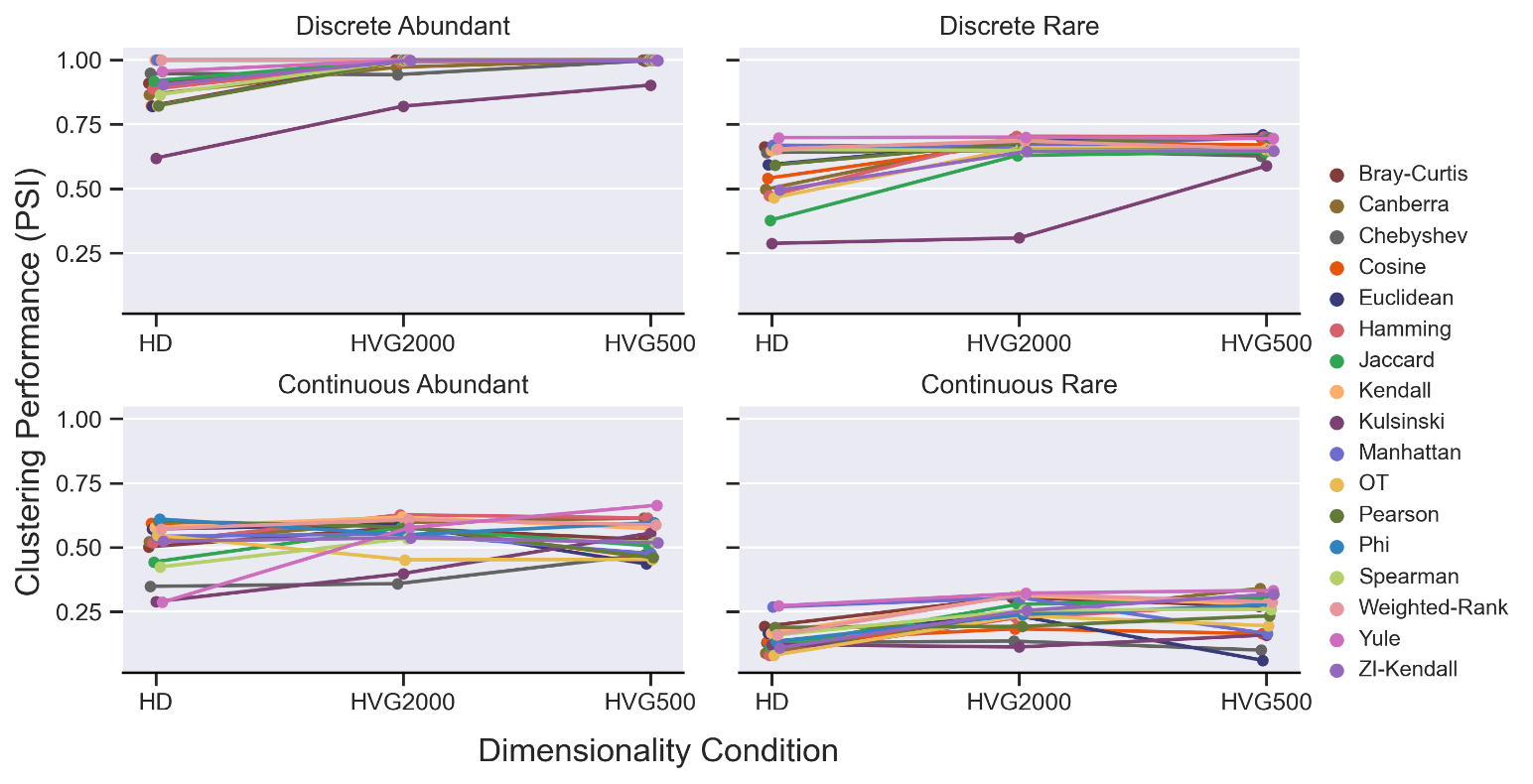


**Figure S4**: Clustering performance of all 17 proximity metrics for each structural condition at high-dimensionality (HD) and two levels of reduced dimensionality based on selection of the top 2000 (HVG2000) and 500 (HVG500) highly variable genes. Points depict the mean Pair Sets Index (PSI) of clustering from neighbourhood sizes of *k* = (3,10,30,50).

**
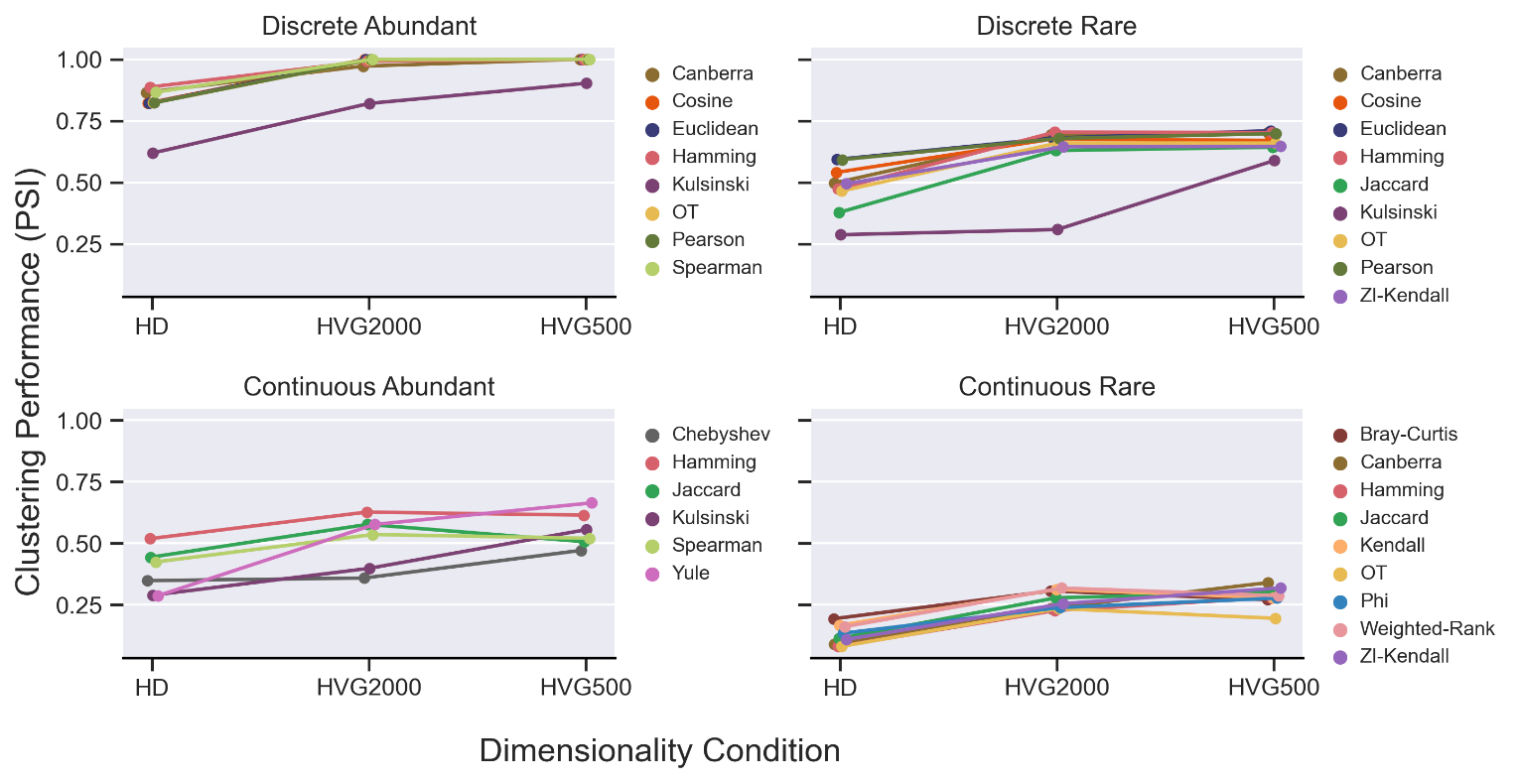
Figure S5:** Clustering performance of the proximity metrics with a >0.1 increase in Pair Sets Index (PSI) change between high-dimensional (HD) dataset and either level of dimensionality reduction (HVG2000, HVG500), for each structural condition. Points depict the mean Pair Sets Index (PSI) of clustering from neighbourhood sizes of *k* = (3,10,30,50).

**
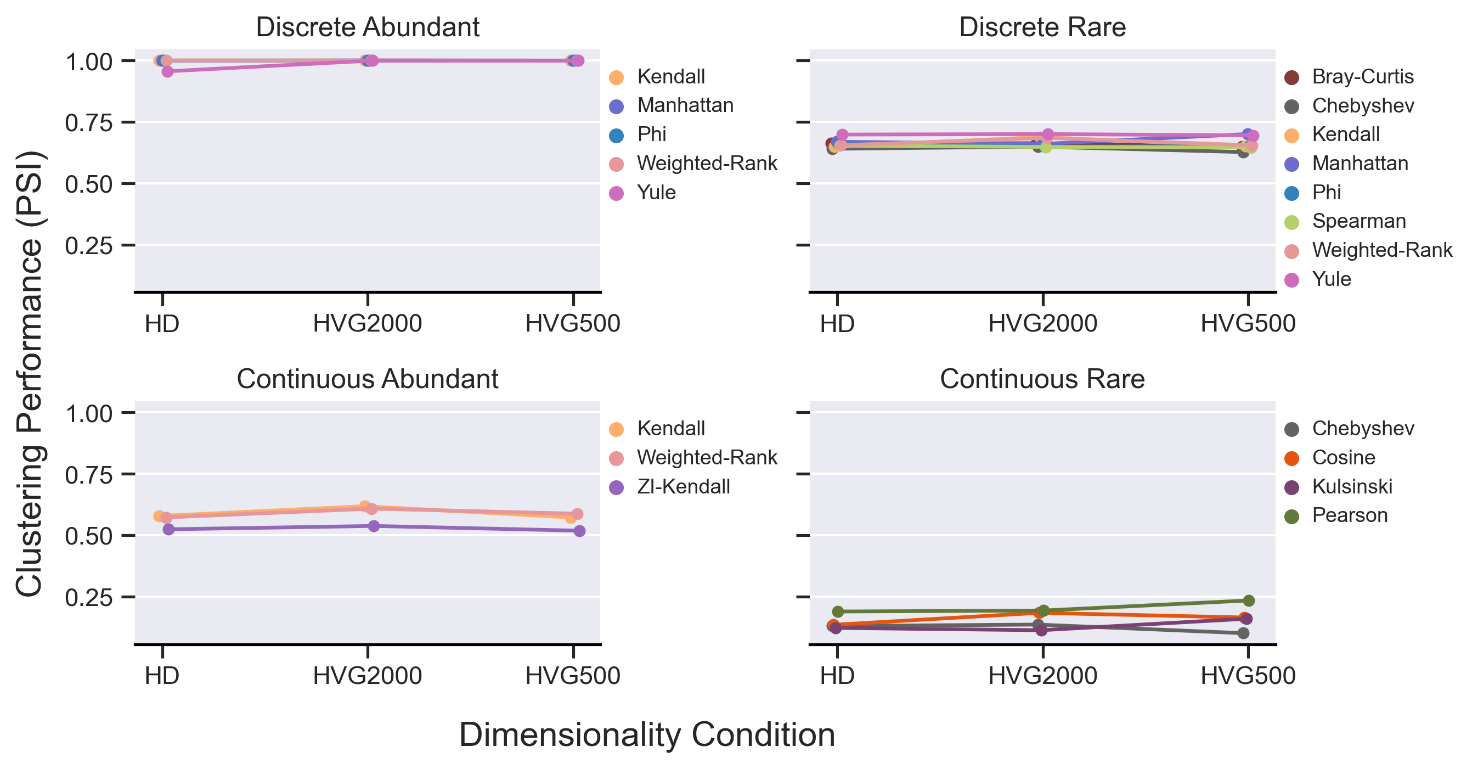
Figure S6**: Performance of all proximity metrics which were identified as invariant, with <0.05 change in between high-dimensional (HD) dataset and either level of dimensionality reduction (HVG2000, HVG500), for each structural condition. Points depict the mean Pair Sets Index (PSI) of clustering from neighbourhood sizes of *k* = (3,10,30,50).


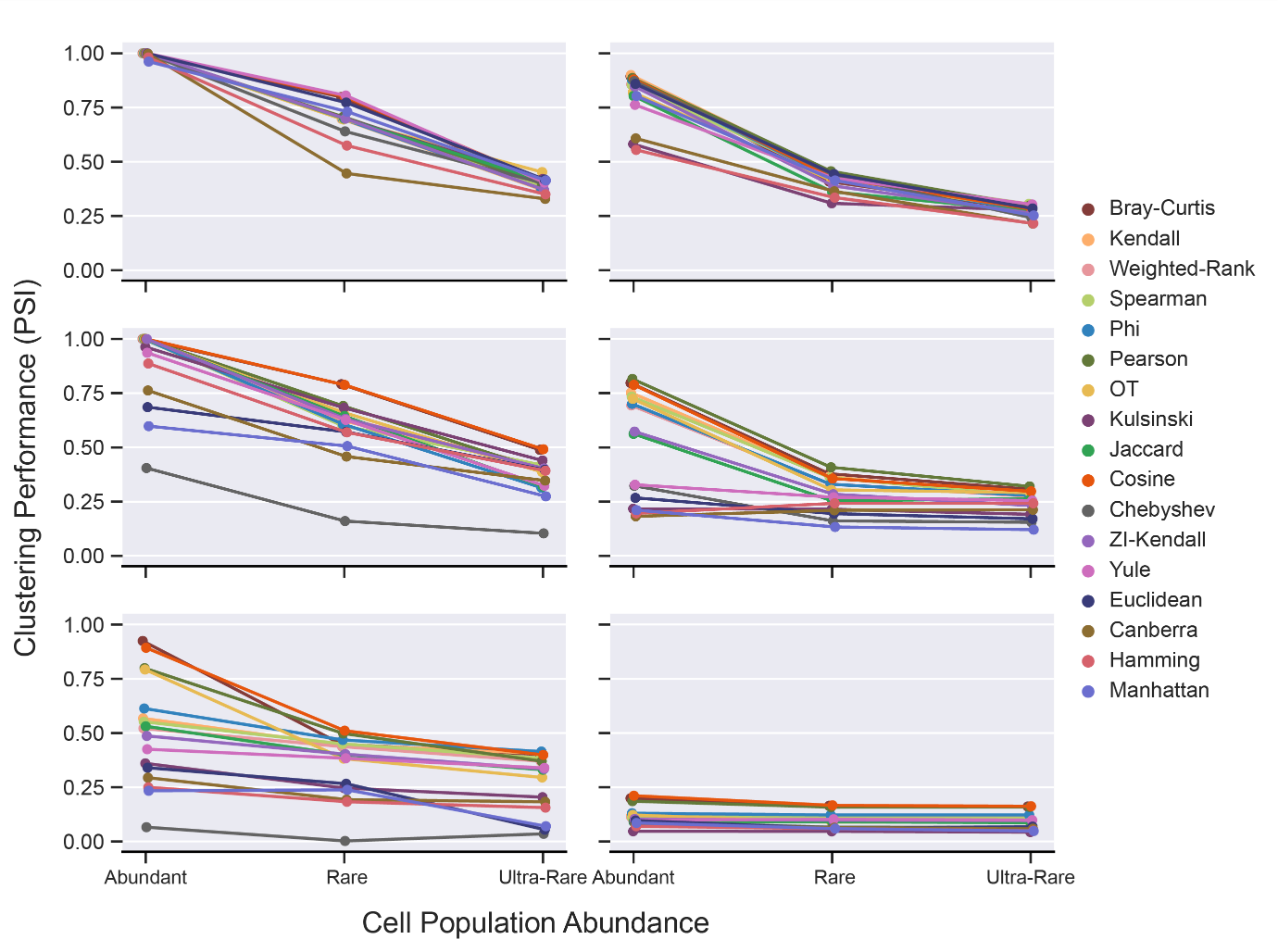
**Figure S7**: Clustering performance of all 17 proximity metrics, for Abundant, Rare, and Ultra-Rare subsets for the Discrete (left) and Continuous (right) simulated scRNA-seq datasets (moderate sparsity). Points depict the mean Pair Sets Index (PSI) of clustering from neighbourhood sizes of *k* = (3,10,30,50).

**
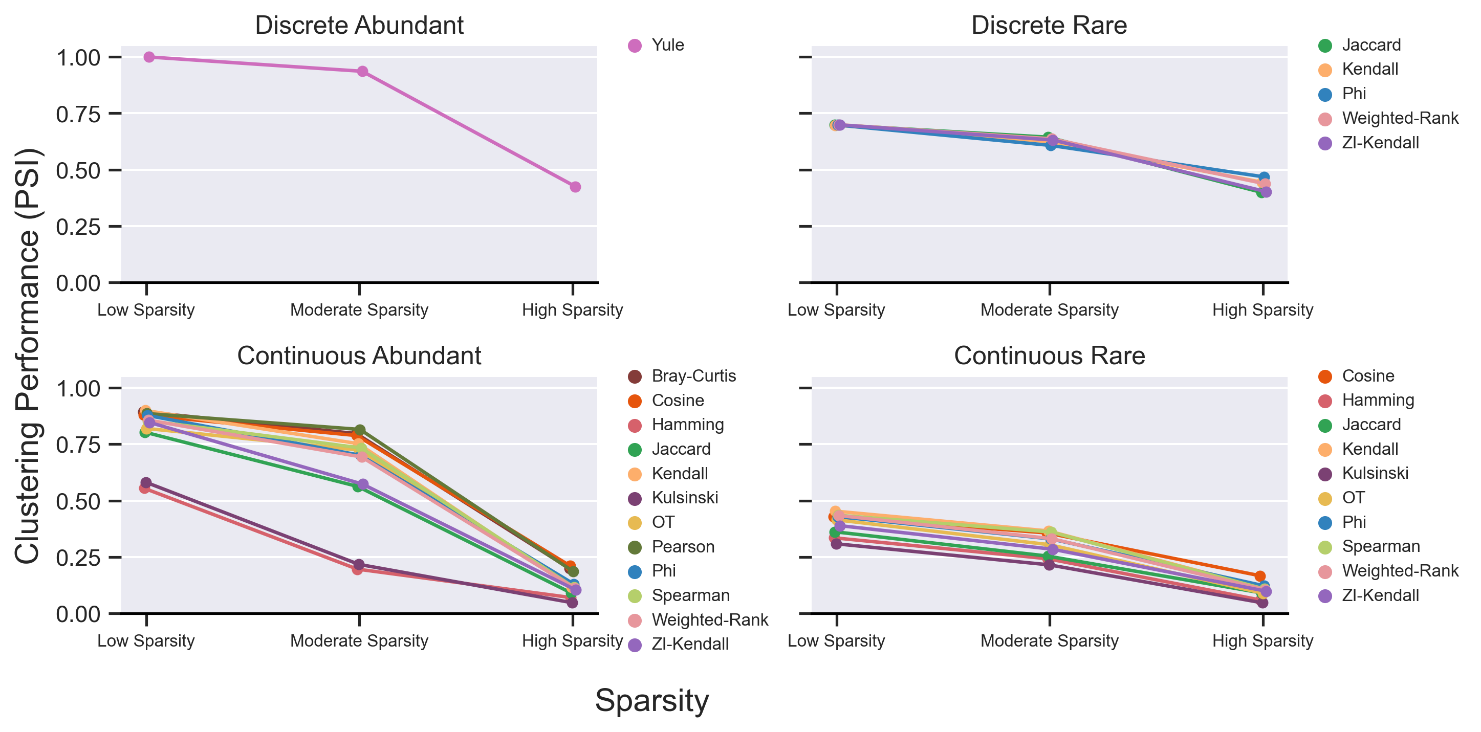
Figure S8**: Performance of proximity metrics identified as moderately sensitive between low (50%) and moderate (70%) sparsity, given a threshold of ≥0.05 but <75th percentile change in PSI. Points depict the mean Pair Sets Index (PSI) of clustering from neighbourhood sizes of *k* = (3,10,30,50).

**
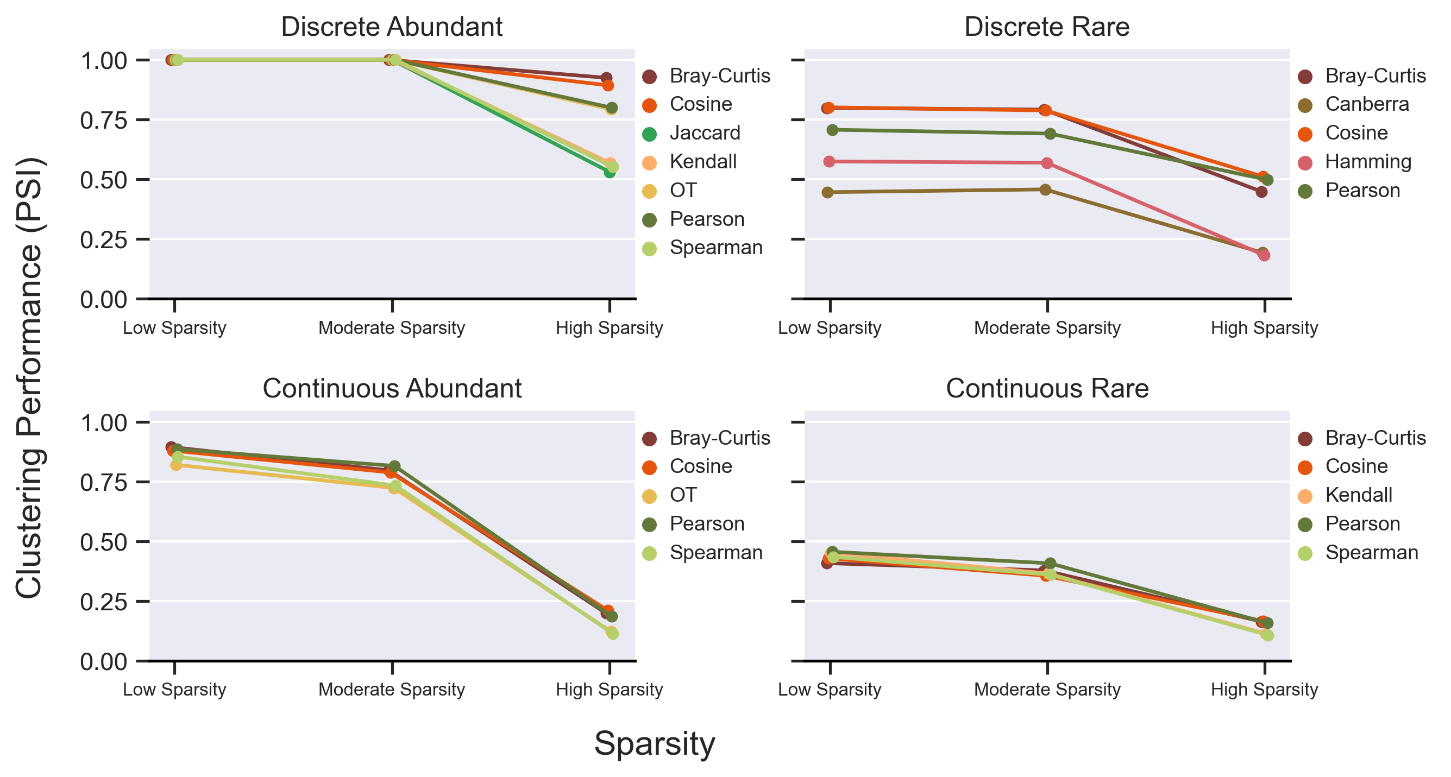
Figure S9**: Performance of the top 5 proximity metrics identified as ranked by smallest change in PSI between low (50%) and moderate (70%) sparsity. Points depict the mean Pair Sets Index (PSI) of clustering from neighbourhood sizes of *k* = (3,10,30,50).

**
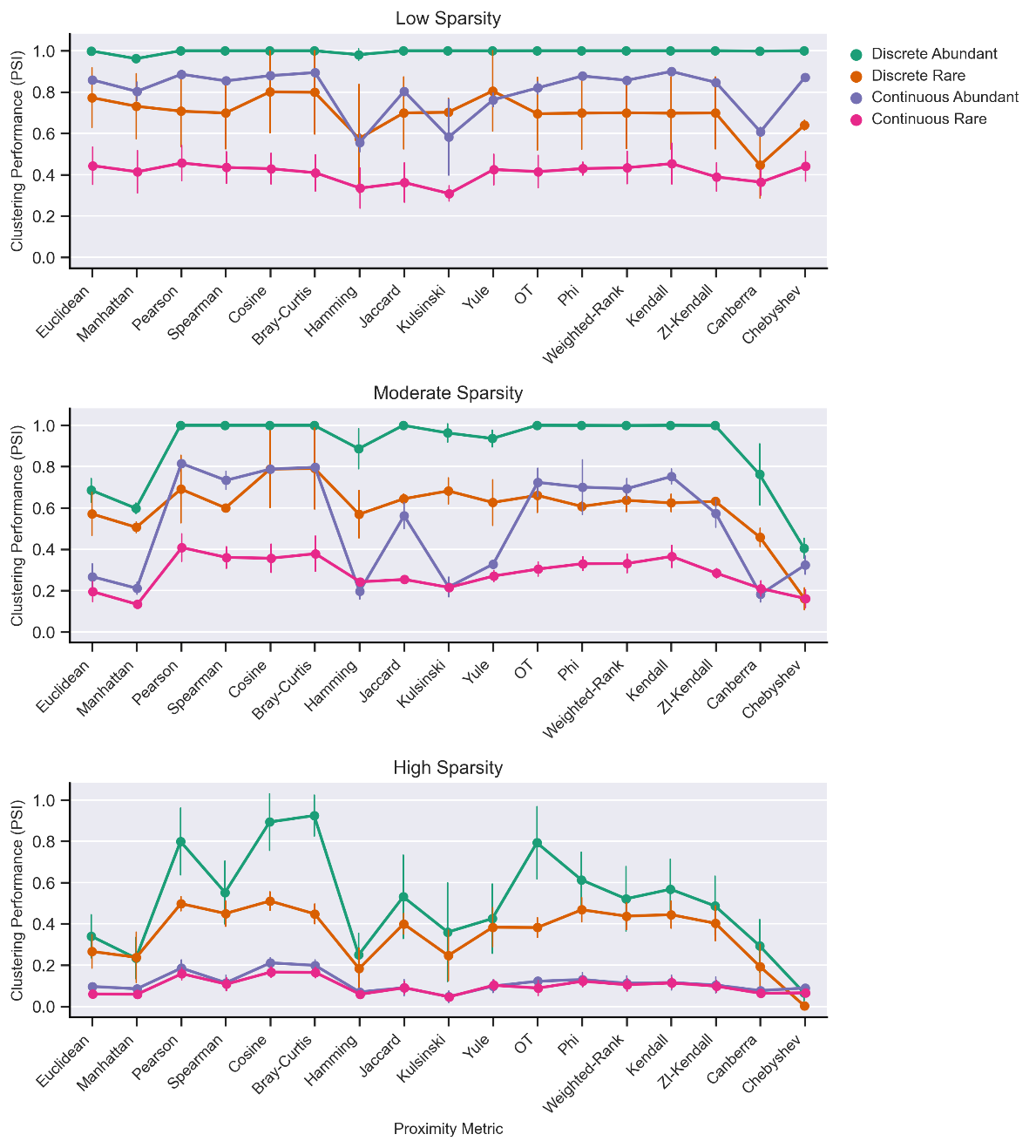
Figure S10**: Clustering performance of all 17 proximity metrics at low (top), moderate (middle) and high (bottom) levels of sparsity, for each structural condition. Points depict the mean Pair Sets Index (PSI) of clustering from neighbourhood sizes of *k* = (3,10,30,50), with error bars depicting one standard deviation.

**Figure S11**: Metric performance across real scRNA-seq datasets of varying structure and **A)** sparsity and **B)** dimensionality. Heatmap cells contain maximum Pair Sets Index (PSI) obtained at a neighbourhood size *k* = 30, for each metric and dataset combination. Heatmap rows are ordered by mean PSI across datasets.


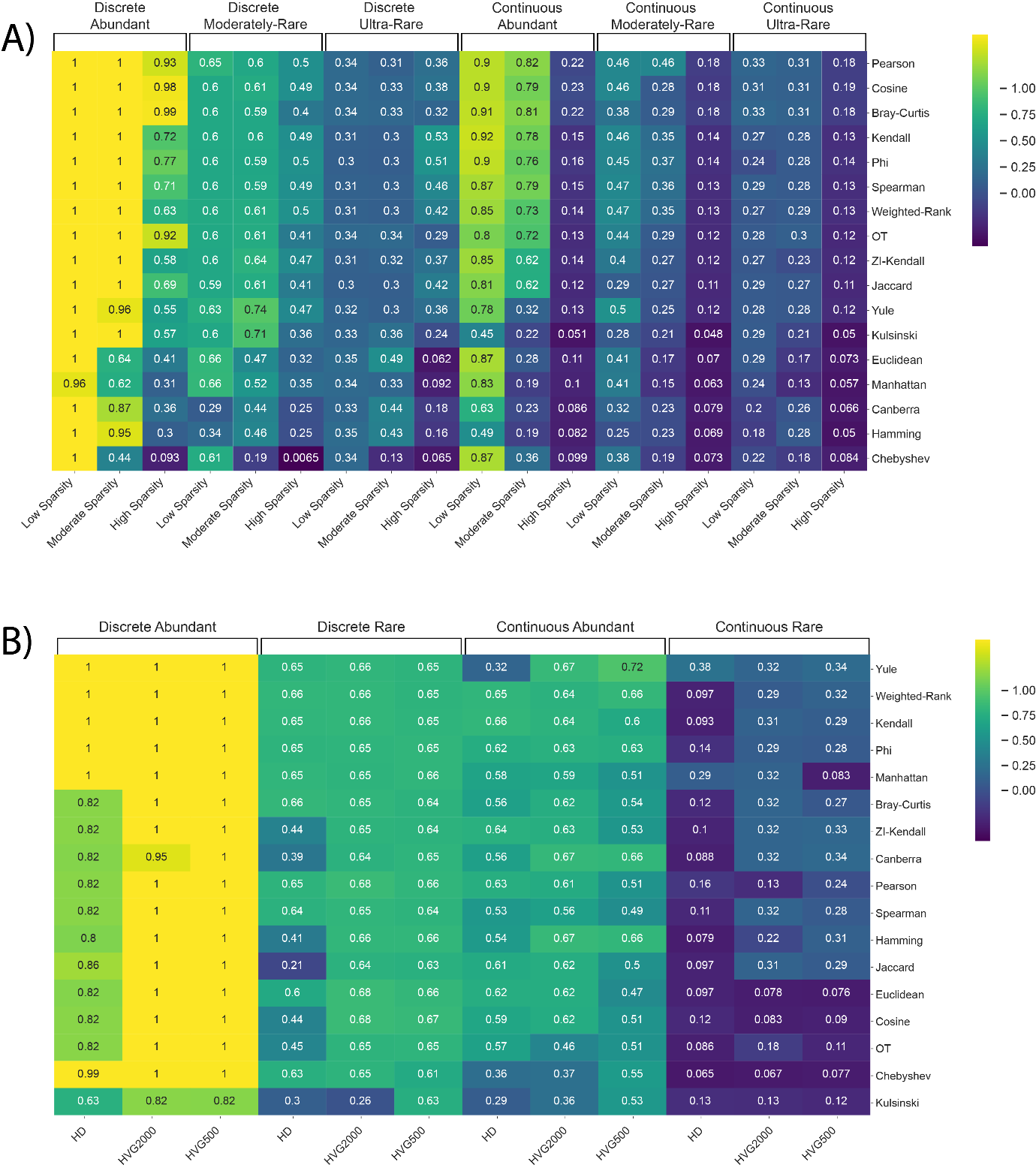

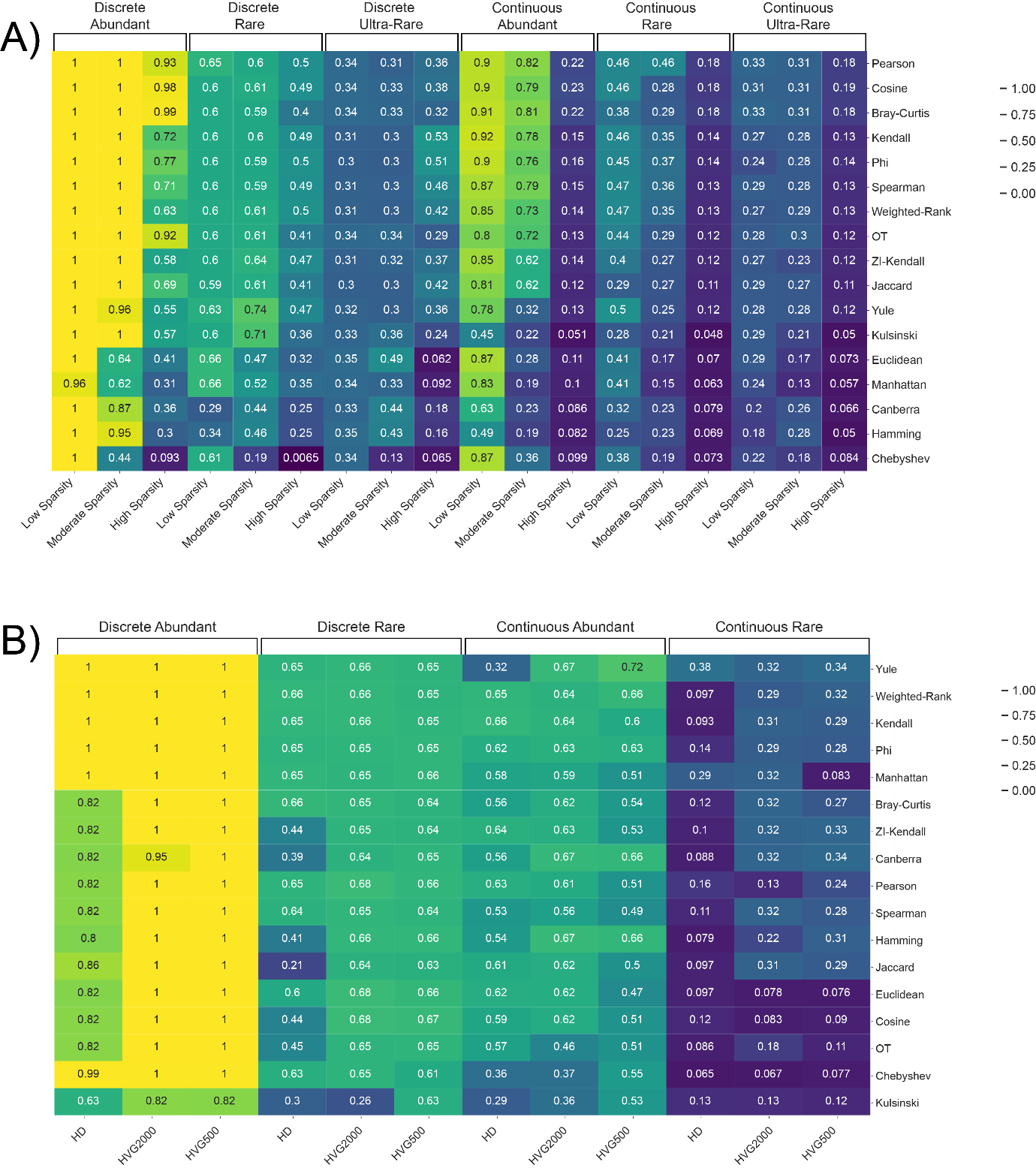


**Figure S12**: Clustering performance (PSI) (mean of n=10 clustering iteration) across neighbourhood size values for KNN, for validation case study datasets of **A)** Discrete-Abundant, **B)** Discrete-Rare, **C)** Continuous-Abundant, and **D)** Continuous-Rare structure. Proximity metrics are included if their maximum PSI across all neighbourhood sizes is ≥ 75th percentile of the maximum performance in the relevant structural class.


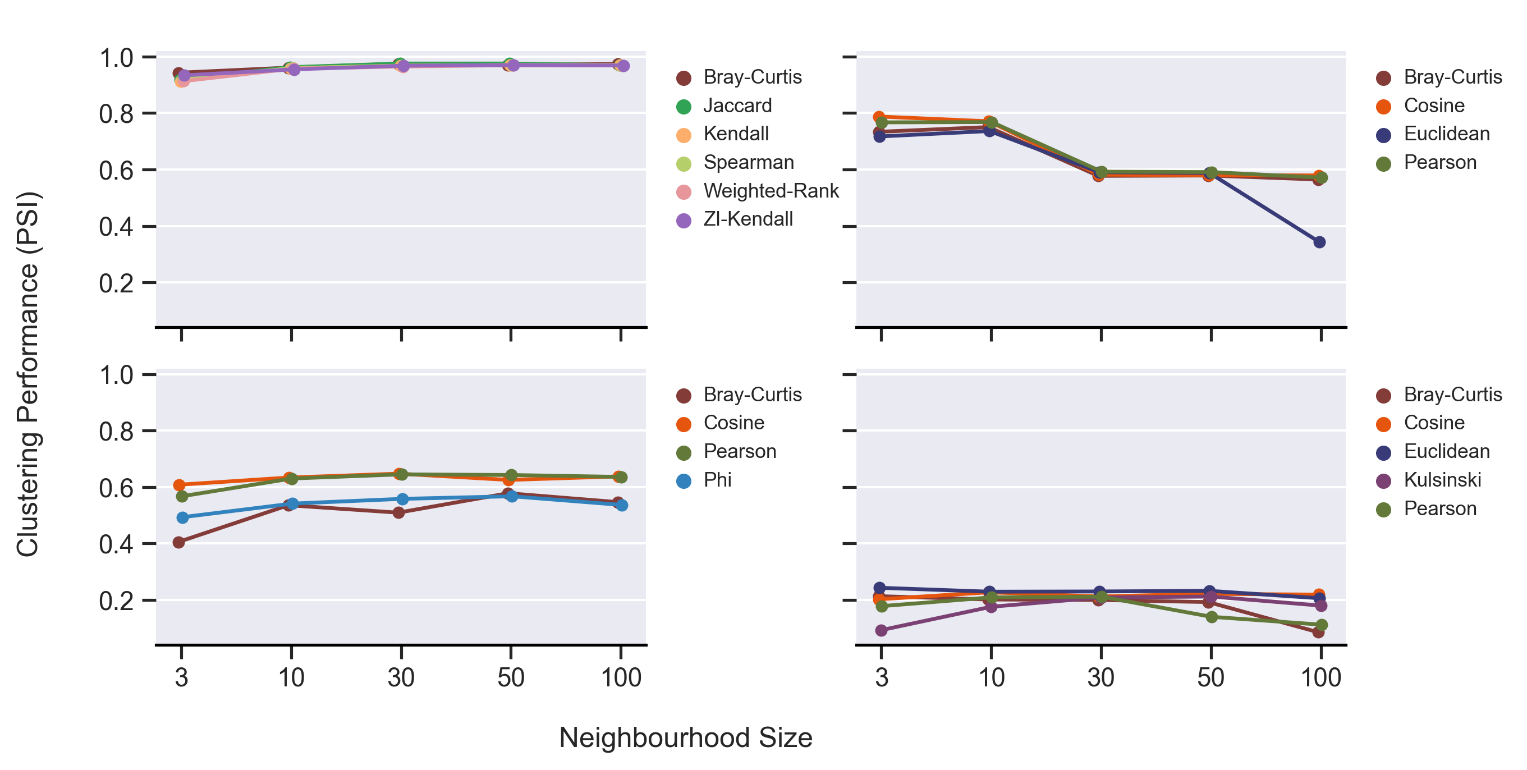


**A)**

**B)**

**C)**

**D)**

# **Supplementary Tables**

**Supplementary Table 1:** Breakdown of cell number and proportion of sub-populations for the CellSIUS [1,2] and the Fetal Liver Haematopoiesis [3,4] datasets.

| Discrete Structure – CellSIUS | | | | | Continuous Structure - Fetal Liver Haematopoiesis | | | | |
| --- | --- | --- | --- | --- | --- | --- | --- | --- | --- |
| Cell Line | Abundant | | Rare | | Cell type | Abundant | | Rare | |
|  | Cell Number | % | Cell Number | % |  | Cell Number | % | Cell Number | % |
| A549 | 400 | 8.0 | 80 | 2.0 | HSC/MPP^1^ | 1000 | 20 | 100 | 2.5 |
| H1437 | 270 | 5.4 | 3 | 0.08 | MEMP^2^ | 1000 | 20 | 3 | 0.075 |
| HCT116 | 1400 | 28.0 | 1599 | 40.1 | Early-erythroid | 1000 | 20 | 2200 | 55 |
| HEK293 | 1600 | 32.0 | 2000 | 50.2 | Mid-erythroid | 1000 | 20 | 1680 | 42 |
| IMR90 | 500 | 10.0 | 100 | 2.5 | Late-erythroid | 1000 | 20 | 17 | 0.425 |
| Jurkat | 100 | 2.0 | 6 | 0.15 | Total | 5000 | 100 | 4000 | 100 |
| K562 | 379 | 7.6 | 70 | 1.8 |  |  |  |  |  |
| Ramos | 350 | 7.0 | 125 | 3.1 |  |  |  |  |  |
| Total | 4999 | 100 | 3983 | 100 |  |  |  |  |  |

^1^Hematopoietic Stem Cell and Multipotent Progenitor

^2^Mega-karyocyte–Erythroid–Mast cell Progenitor

**Supplementary Table 2:** Breakdown of cell number and proportion of sub-populations for the simulated scRNA-seq datasets [13] of Discrete and Continuous structure, subset for Abundant, Rare and Ultra-Rare populations. T = Trajectory of Differentiation, B = branch segment of the differentiation trajectory.

| Discrete – Simulated data | | | | | | | Continuous - Simulated data | | | | | | |
| --- | --- | --- | --- | --- | --- | --- | --- | --- | --- | --- | --- | --- | --- |
| Cell-type | Abundant | | Rare | | Ultra-Rare | | Cell-type | Abundant | | Rare | | Ultra-Rare | |
|  | Cell *n* | % | Cell *n* | % | Cell *n* | % |  | Cell *n* | % | Cell *n* | % | Cell *n* | % |
| Origin | 500 | 20 | 10 | 1 | 3 | 0.3 | Origin | 500 | 7.69 | 400 | 40 | 500 | 50 |
| T1, B3 | 500 | 20 | 30 | 3 | 7 | 0.7 | T1 B1 | 500 | 7.69 | 110 | 11 | 115 | 11.5 |
| T2, B3 | 500 | 20 | 160 | 16 | 40 | 4 | T1, B2 | 500 | 7.69 | 30 | 3 | 7 | 0.7 |
| T3, B3 | 500 | 20 | 300 | 30 | 450 | 45 | T1, B3 | 500 | 7.69 | 10 | 1 | 3 | 0.3 |
| T4, B3 | 500 | 20 | 500 | 50 | 500 | 50 | T2, B1 | 500 | 7.69 | 10 | 1 | 3 | 11.5 |
| *Total* | 2500 | 100 | 1000 | 100 | 1000 | 100 | T2, B2 | 500 | 7.69 | 110 | 11 | 115 | 0.7 |
|  |  |  |  |  |  |  | T2, B3 | 500 | 7.69 | 30 | 3 | 7 | 0.3 |
|  |  |  |  |  |  |  | T3, B1 | 500 | 7.69 | 30 | 3 | 7 | 11.5 |
|  |  |  |  |  |  |  | T3, B2 | 500 | 7.69 | 10 | 1 | 3 | 0.7 |
|  |  |  |  |  |  |  | T3, B3 | 500 | 7.69 | 110 | 11 | 115 | 0.3 |
|  |  |  |  |  |  |  | T3, B1 | 500 | 7.69 | 110 | 11 | 115 | 11.5 |
|  |  |  |  |  |  |  | T3, B2 | 500 | 7.69 | 10 | 1 | 3 | 0.7 |
|  |  |  |  |  |  |  | T3, B3 | 500 | 7.69 | 30 | 3 | 7 | 0.3 |
|  |  |  |  |  |  |  | *Total* | 6500 | 100 | 1000 | 100 | 1000 | 100 |

**Supplementary Table 3:** Details of percentage of sparsity and cell and gene number for each of the simulated scRNA-seq datasets, Pre- and Post-processing.

| **Sparsity:** | Low Sparsity | | | | Moderate Sparsity | | | High Sparsity | | |
| --- | --- | --- | --- | --- | --- | --- | --- | --- | --- | --- |
| **Processing:** | Pre | | Post | | Pre | Post | | Pre | Post | |
| **Condition** | Cell x Gene | % | % | Cell x Gene | % | % | Cell x Gene | % | % | Cell x Gene |
| Discrete  Abundant | 2500 × 5000 | 49.8 | 46.8 | 2500 × 4691 | 71 | 67.8 | 2500 × 4691 | 90 | 85.2 | 2474 × 2412 |
| Discrete Rare | 1000 × 5000 | 49.4 | 45.6 | 1000 × 4622 | 69.9 | 67.1 | 1000 × 4622 | 89.9 | 85 | 991 × 2473 |
| Discrete  Ultra-Rare | 1000 × 5000 | 48.8 | 44.7 | 1000 × 4593 | 70.5 | 66.4 | 1000 × 4593 | 89.8 | 84.8 | 992 × 2447 |
| Continuous Abundant | 6500 × 5000 | 48.3 | 45 | 6500 × 4669 | 70.1 | 66.7 | 6500 × 4669 | 89.7 | 85 | 6452 × 2537 |
| Continuous Rare | 1000 × 5000 | 46.5 | 43.5 | 1000 × 4713 | 69.1 | 65.9 | 1000 × 4713 | 89.3 | 85 | 990 × 2700 |
| Continuous  Ultra-Rare | 1000 × 5000 | 46.1 | 43.1 | 1000 × 4714 | 68.9 | 65.7 | 1000 × 4714 | 68.8 | 84.9 | 991 × 2703 |

**Supplementary Table 4**: Cell number, gene number and dataset sparsity before and after processing for the CellSIUS [1,2] and the Fetal Liver Haematopoiesis (FLH) [3,4] datasets.

| **Dataset** | **Structural Condition** | **Pre-Processing** | | **Post-Processing** | |
| --- | --- | --- | --- | --- | --- |
|  |  | Cell x Gene | Sparsity (%) | Cell x Gene | Sparsity (%) |
| CellSIUS | Discrete Abundant | 5000 × 23848 | 84.9 | 5000 × 8982 | 63.1 |
| CellSIUS | Discrete Rare | 3984 × 23848 | 85.1 | 3984 × 8802 | 63.1 |
| FLH | Continuous Abundant | 5000 × 27080 | 87.8 | 5000 × 8174 | 62.4 |
| FLH | Continuous Rare | 4000 × 27080 | 85.1 | 4000 × 8622 | 55.4 |

**Supplementary Table 5:** Implementation details for the 17 proximity metrics investigated. All data underwent filtering and normalisation prior to calculation of proximity metric matrices. If the output from a proximity metric function was not in the form of a dissimilarity metric, additional computations were performed to convert them.

| **Metric** | **Class** | **Input** | **Language** | **Package** |
| --- | --- | --- | --- | --- |
| Euclidean | Distance | Counts | Python (v3.8) [9] | sklearn (v1.0.1) [16] |
| Manhattan | Distance | Counts | Python (v3.8) [9] | sklearn (v1.0.1) [16] |
| Canberra | Distance | Counts | Python (v3.8) [9] | SciPy (v1.7.1)[14] |
| Chebyshev | Distance | Counts | Python (v3.8) [9] | SciPy (v1.7.1)[14] |
| Hamming | Correlation | Binarised | Python (v3.8) [9] | SciPy (v1.7.1)[14] |
| Pearson | Correlation | Counts | Python (v3.8) [9] | SciPy (v1.7.1)[14] |
| Spearman^1^ | Correlation | Counts | Python (v3.8) [9] | SciPy (v1.7.1)[14] |
| Weighted-Rank^1^ | Correlation | Counts | R (v4.1.1) [5] | dismay [15] |
| Kendall^1^ | Correlation | Counts | R (v4.1.1) [5] | dismay [15] |
| ZI-Kendall^1,2^ | Correlation | Counts | R (v4.1.1) [5] | dismay [15] |
| Bray-Curtis | Proportionality | Counts | Python (v3.8) [9] | SciPy (v1.7.1)[14] |
| Cosine | Similarity | Counts | Python (v3.8) [9] | SciPy (v1.7.1)[14] |
| Phi^3^ | Proportionality | Counts | R (v4.1.1) [5] | dismay [15] |
| Optimal Transport^4^ | Dissimilarity | Counts | Python (v3.8) [9] | Otscomics (v0.0.1)[22] |
| Jaccard index | Dissimilarity | Binarised | Python (v3.8) [9] | SciPy (v1.7.1)[14] |
| Kulsinski | Dissimilarity | Binarised | Python (v3.8) [9] | SciPy (v1.7.1)[14] |
| Yule | Dissimilarity | Binarised | Python (v3.8) [9] | SciPy (v1.7.1)[14] |

^1^To transform the outputted cellxcell matrix values into the form of a dissimilarity, all matrix values were subtracted from 1.

^2^Rescaled outputted cellxcell matrix to contain values in a bounded range (0 – 1) and enforced a 0 diagonal.

^3^To transform the outputted cellxcell matrix values into the form of a dissimilarity, the absolute values of outputted cellxcell matrix were taken.

^4^Requires GPU.

**Supplementary Table 6**: Representative datasets and their respective performance ranges for the recommended proximity metrics and neighbourhood sizes (*k*) structural properties (Figure 9). As simulated scRNA-seq data was used to evaluate the influence of sparsity, these datasets generally contain lower levels of noise and complexity as real scRNA-seq data. As such, higher PSI ranges are typically observed for the simulated datasets.

| **Structure** | **Dataset** | **Properties** | **Proximity Measures** | ***k*** | **PSI^2^ range** |
| --- | --- | --- | --- | --- | --- |
| Discrete Abundant | CellSIUS[1] | Reduced dimensionality | Any metric but Kulsinski | Any | PSI >0.99 |
|  | Simulated[13] | High Sparsity | Bray-Curtis, Cosine | 30,50,100 | PSI > 0.98 |
|  |  | Low/Moderate Sparsity | Weighted Rank, Kendall, Phi | Any | PSI >0.99 |
| Discrete Rare | CellSIUS[1] | Reduced dimensionality | Euclidean, Manhattan, Yule, Hamming, Canberra, Pearson, Cosine | 3 | 0.67 < PSI < 0.71 |
|  | Simulated[13] | High Sparsity | Phi, Spearman, Kendall | 100 | 0.54 < PSI < 0.64 |
|  |  |  | Pearson, Cosine | 3 |  |
|  |  | Low/Moderate Sparsity | Bray-Curtis, Pearson, Cosine | 3 | 0.67 < PSI < 0.71 |
| Continuous Abundant | FLH^1^[3] | Reduced dimensionality | Yule, Kendall, Weighted-Rank | 3,10 | 0.28 < PSI < 0.34 |
|  | Simulation[13] | High Sparsity | Bray-Curtis, Pearson, Cosine | 30,50,100 | 0.18 < PSI < 0.2 |
|  |  | Low/Moderate Sparsity | Bray-Curtis, Kendall, Pearson | 3,10 | 0.41 < PSI < 0.48 |
| Continuous Rare | FLH^1^[3] | Reduced dimensionality | Yule, Hamming, Canberra, Kendall, Weighted-Rank | Any | 0.57 < PSI < 0.67 |
|  | Simulated[13] | High Sparsity | Bray-Curtis, Pearson, Cosine | 30,50,100 | 0.18 < PSI <0.24 |
|  |  | Low/Moderate Sparsity | Bray-Curtis, Pearson, Cosine | 30,50,100 | 0.76 < PSI < 0.83 |

^1^Fetal Liver Haematopoiesis.

^2^Pair Sets Index.

**Supplementary Table 7**: Breakdown of cell number and proportion of sub-populations for the case-study scRNA-seq datasets selected to validate recommendations in Figure 9. See Supplementary 4: Validation Analysis for further details.

| Discrete Abundant | | | Discrete Rare | | | Continuous Abundant | | | Continuous Rare | | |
| --- | --- | --- | --- | --- | --- | --- | --- | --- | --- | --- | --- |
| Type | Num. | % | Type | Num. | % | Type | Num. | % | Type | Num. | % |
| BC2^1^ | 425 | 21.25 | SMC^4^ | 938 | 47.1 | MSC^7^ | 565 | 30 | Neural Crest | 1000 | 50 |
| BC6^1^ | 375 | 18.75 | Fibroblast 1b | 594 | 29.82 | Adipocyte progenitor | 456 | 24.2 | Keratocyte I | 825 | 41.25 |
| BC7^1^ | 325 | 16.25 | LEC^5^ | 310 | 15.56 | Osteoblast progenitor | 328 | 17.41 | Keratocyte II | 5 | 0.25 |
| BC4^1^ | 310 | 15.5 | ICC 1^6^ | 75 | 3.76 | Pro-osteoblast | 327 | 17.36 | Keratocyte III | 20 | 1 |
| RBC^2^ | 290 | 14.5 | Glial | 50 | 2.51 | Pre-adipocyte | 208 | 11.04 | Fibroblast I | 50 | 2.5 |
| MG^3^ | 275 | 13.75 | MSC^7^ | 20 | 1 | Total | 1,884 | 100 | Fibroblast II | 100 | 5 |
| Total | 2000 | 100 | Pericytes | 5 | 0.25 |  |  |  | Total | 2000 | 100 |
|  |  |  | Total | 1992 | 100 |  |  |  |  |  |  |

^1^BC = Cone Bipolar Cells

^2^Rod Bipolar Cells

^3^Mueller Glia

^4^Smooth Muscle Cells (type 1).

^5^Lymphatic Endothelial Cells

^6^Interstitial Cells of Cajal (type 1)

^7^Meschenchymal Stem Cells

**^Supplementary References^**

1. Wegmann R, Neri M, Schuierer S, et al. CellSIUS provides sensitive and specific detection of rare cell populations from complex single-cell RNA-seq data. Genome Biology 2019; 20:142

2. Wegmann R, Neri M. CellSIUS provides sensitive and specific detection of rare cell populations from complex single cell RNA-seq data: Codes and processed data. 2019;

3. Popescu D-M, Botting RA, Stephenson E, et al. Decoding human fetal liver haematopoiesis. Nature 2019; 574:365–371

4. Popescu D-M, Botting RA, Stephenson E, et al. Decoding human fetal liver haematopoiesis: Dataset. 2019;

5. R Core Team. R: The R Project for Statistical Computing. 2021;

6. Hao Y, Hao S, Andersen-Nissen E, et al. Integrated analysis of multimodal single-cell data. Cell 2021; 184:3573-3587.e29

7. Virshup I, Rybakov S, Theis FJ, et al. anndata: Annotated data. 2021; 2021.12.16.473007

8. Cakir B, Prete M, Huang N, et al. Comparison of visualization tools for single-cell RNAseq data. NAR Genom Bioinform 2020; 2:lqaa052

9. Van Rossum G, Drake FL. Python 3 Reference Manual. 2009;

10. Wolf FA, Angerer P, Theis FJ. SCANPY: large-scale single-cell gene expression data analysis. Genome Biology 2018; 19:15

11. Watson ER, Mora A. scProximitE. 2022;

12. Watson ER, Mora A, Taherian Fard A, et al. Evaluating the influence of structural properties on proximity metric performance in single cell RNA-seq data - Datasets (0.0.1) [Data set]. 2022;

13. Papadopoulos N, Gonzalo PR, Söding J. PROSSTT: probabilistic simulation of single-cell RNA-seq data for complex differentiation processes. Bioinformatics 2019; 35:3517–3519

14. Virtanen P, Gommers R, Oliphant TE, et al. SciPy 1.0: fundamental algorithms for scientific computing in Python. Nat Methods 2020; 17:261–272

15. Skinnider MA, Squair JW, Foster LJ. Evaluating measures of association for single-cell transcriptomics. Nat Methods 2019; 16:381–386

16. Pedregosa F, Varoquaux G, Gramfort A, et al. Scikit-learn: Machine Learning in Python. Journal of Machine Learning Research 2011; 12:2825–2830

17. Traag V, Waltman L, van Eck NJ. From Louvain to Leiden: guaranteeing well-connected communities. Sci Rep 2019; 9:5233

18. Gagolewski M. genieclust: Fast and robust hierarchical clustering. SoftwareX 2021; 15:100722

19. Rezaei M, Fränti P. Set Matching Measures for External Cluster Validity. IEEE Transactions on Knowledge and Data Engineering 2016; 28:2173–2186

20. Steinley D. Properties of the Hubert-Arabie adjusted Rand index. Psychol Methods 2004; 9:386–396

21. Vinh NX, Epps J, Bailey J. Information Theoretic Measures for Clusterings Comparison: Variants, Properties, Normalization and Correction for Chance. 18

22. Huizing G-J, Peyré G, Cantini L. Optimal Transport improves cell-cell similarity inference in single-cell omics data. 2021; 2021.03.19.436159

23. Shekhar K, Lapan SW, Whitney IE, et al. Comprehensive Classification of Retinal Bipolar Neurons by Single-Cell Transcriptomics. Cell 2016; 166:1308-1323.e30

24. Jasso GJ, Jaiswal A, Varma M, et al. Colon stroma mediates an inflammation-driven fibroblastic response controlling matrix remodeling and healing. PLOS Biology 2022; 20:e3001532

25. Wolock SL, Krishnan I, Tenen DE, et al. Mapping Distinct Bone Marrow Niche Populations and Their Differentiation Paths. Cell Reports 2019; 28:302-311.e5

26. Collin J, Queen R, Zerti D, et al. A single cell atlas of human cornea that defines its development, limbal progenitor cells and their interactions with the immune cells. The Ocular Surface 2021; 21:279–298

27. Hafemeister C, Satija R. Normalization and variance stabilization of single-cell RNA-seq data using regularized negative binomial regression. Genome Biology 2019; 20:296
